# The Structures Obtained from the Oxidation of 3‑Amino‑1*H*‑indazole in Basic Conditions According to Hünig and Pozharskii

**DOI:** 10.1021/acs.joc.6c00198

**Published:** 2026-06-17

**Authors:** Hélio M. T. Albuquerque, Luís F. B. Fontes, Samuel Guieu, Artur M. S. Silva, Ibon Alkorta, José Elguero

**Affiliations:** † LAQV-REQUIMTE & Department of Chemistry, 56062 Universidade de Aveiro, Aveiro 3810-193, Portugal; ‡ CICECO & Department of Chemistry, Universidade de Aveiro, Aveiro 3810-193, Portugal; § Instituto de Química Médica, CSIC, Juan de la Cierva, 3, E-28006 Madrid, Spain

## Abstract

The oxidation of 3-amino-1*H*-indazole was reported independently by Hünig and Pozharskii approximately 50 years ago and proposed to yield an azo compound via a Baeyer–Mills-type mechanism. However, the structural assignment lacked complete evidence, leaving the identity of the obtained product uncertain. To unambiguously determine the correct structure, the oxidation under basic conditions was reinvestigated, revealing two distinct products depending on the loading of *t*-BuLi. The obtained compounds were systematically characterized by 1D/2D NMR techniques and X-ray diffraction, leading to the conclusion that the Hünig′s “deep-red” compound corresponds to the structure assigned as (*E*)-1-[(3*H*-indazo-3-ylidene)­amino]-1*H*-3-amine, whereas Pozharskii′s “orange” compound is indeed an azoazole, featuring an *E*-configuration in the azo bond rather than the *Z*-configuration reported in 1977. This investigation resolves discrepancies among previous literature reports regarding the oxidation of 3-amino-1*H*-indazole and unifies them within a single coherent framework.

## Graphical abstract

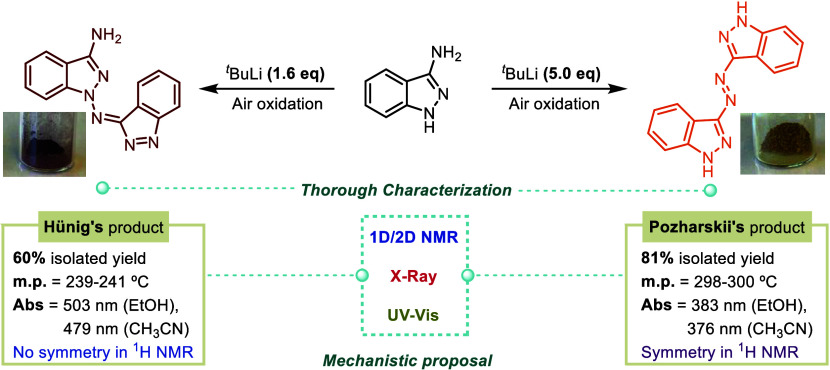

## Introduction

The azo bonds in general and especially in azobenzenes are well-known for their ability to modify their configuration using thermal and/or electromagnetic energy. These compounds are recognized as molecular switches.^1–3^ Photoswitchable compounds have a wide range of potential applications, including photopharmacology,^4^ optochemical genetics, and data storage. Among these, azobenzenes are particularly well-studied due to their ease of synthesis and high stability. Photoinduced isomerization of azobenzenes results in significant changes to their shape and length, a feature that has been leveraged to tune the folding and unfolding of peptides and proteins.^5,6^

Numerous types of photoswitches have been developed for applications in both material science and biology. A well-studied example is the azobenzene photoswitch, which absorbs light most strongly at about 320 nm and undergoes (*E*) to (*Z*)-photoisomerization under 365 nm light.^7,8^ The reverse isomerization, from (*Z*) to (*E*), can occur either thermally or photochemically. Recently, azophotoswitches containing heteroaryl units have attracted increasing attention because of their unique light absorption and photoisomerization properties. Notably, photoswitches with five-membered heteroaryl units exhibit distinct photophysical and structural properties (Figure 1).^9^

**Figure 1:**
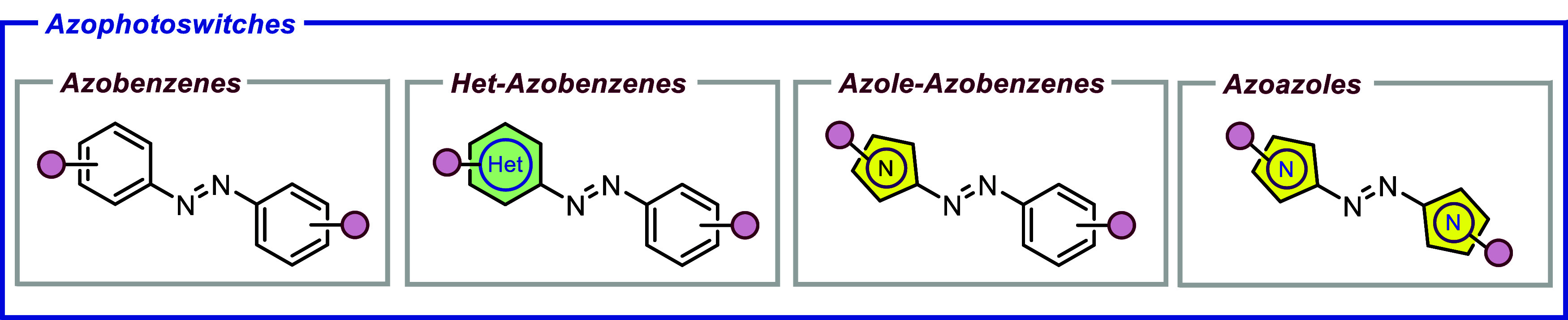
Representation of azophotoswitches and some of their subgroups.

Azo derivatives of azoles (Az) are of particular interest as photoswitches, typically exhibiting an Az-N=N–Ar structure (where Ar represents a phenyl group, another aryl group, or an aromatic heterocycle different from the azole, such as thiophene or thiazole) (Figure 1).^10–13^ A subclass of these compounds features the Az1-N=N-Az2 structure (Figure 1). Considering only the parent compounds, there are 780 compounds in this category, 39 of which are symmetric and 741 asymmetric.

Upon synthesis, all NH-azoazoles typically adopt the (*E*)-configuration of the most stable tautomer (in the case of indazole, the 1*H*). The only exception is a 1976 study by Hünig and Steinmetzer.^14^ In this study, upon oxidizing 3-amino-1*H*-indazole under basic conditions, the authors obtained a red product with the formula C_14_H_10_N_6_, which they concluded was not the “classical” (*E*)-structure, but rather an azo compound with (*Z*)-stereochemistry or a hydrazone tautomer with an intramolecular hydrogen bond (IMHB) (Figure 2A). Both are interesting compounds; the ground state (*Z*) of azo compounds is unknown, and azo/hydrazo tautomerism, although known for other azo derivatives,^15–18^ is unknown for Az1-N=N-Az2 compounds. Regarding the characterization of these compounds, the scarce information provided by Hünig is reported in Figure 2B.

**Figure 2:**
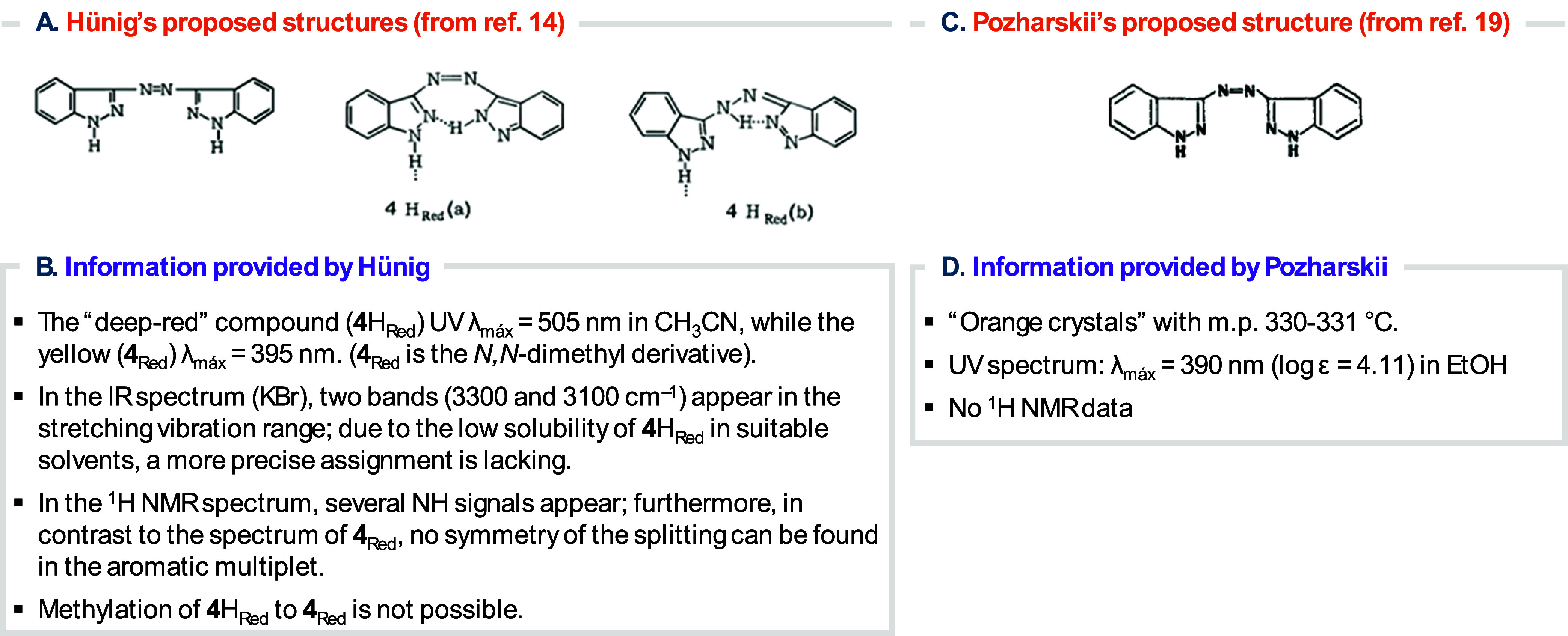
Available literature data on the structures and properties of the azoazoles reported by Hünig and Pozharskii.

Almost simultaneously, Filipskikh and Pozharskii also reported the formation of an azo compound via oxidation of 3-amino-1*H*-indazole under basic conditions, proposing a structure identical to one of those put forward by Hünig (Figure 2C).^19^ Likewise, the available information relating to the characterization of the compound is rather insufficient. The reported compound, crystallized in ethanol-dimethylformamide, was described as orange in color and absorbing at 390 nm (log ε = 4.11) in EtOH (Figure 2D). Despite the similar UV band at ∼390 nm reported by both authors and assigned to a methylated compound by Hünig, we noticed that Hünig′s compound **4**H_Red_ exhibited an additional band at 505 nm in acetonitrile (Figure 2B). But the information that bewildered us the most was the inexistence of symmetry in the ^1^H NMR of the compound reported by Hünig, raising questions about its structural assignment.

Considering the discrepancies and a certain degree of ambiguity between the data reported by the two studies, we undertook a detailed investigation of the oxidation of 3-amino-1*H*-indazole under basic conditions. The resulting compounds were systematically characterized using modern techniques, including 1D and 2D NMR spectroscopy and X-ray diffraction, complemented by UV–vis and FTIR analyses to enable direct comparison with the literature data from 1976 to 77.

## Results and Discussion

We began our investigations by minimizing the energy of the structures for the (*E*)-azo **1**, the (*Z*)-azo **2**, and hydrazone **3**, as depicted in Figure 3. The optimized geometry of the (*E*)-azo isomer **1** corresponds to a relative energy of 0.0 kJ·mol^–1^ in the gas phase, whereas the (*Z*)-azo isomer **2** and the hydrazone form **3** are significantly higher in energy, at 51.7 and 34.1 kJ·mol^–1^, respectively. These results suggest that both the *Z*-configuration and the hydrazone form should be relatively unstable, making them unlikely candidates for the structures reported by Hünig and Pozharskii.

**Figure 3:**
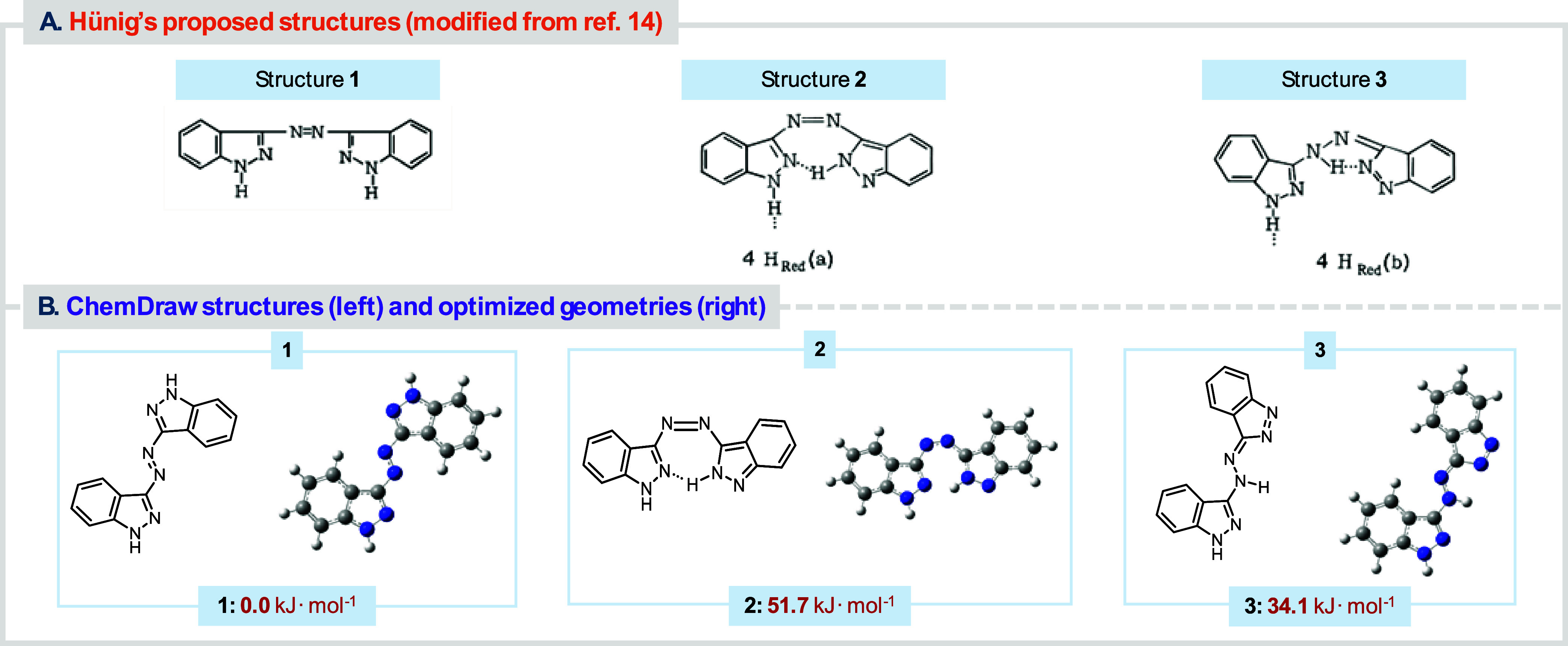
(A) The 3 isomers of Hünig′s compound.^14^ The numbering in brackets with “Red” refers to “reduzierte”. (B) The relative energies in the gas phase of compounds **2** and **3** compared to compound **1** are also given. Structure **2** is also called **4**H_Red_(a), and structure **3** is also called **4**H_Red_(b).

These preliminary results were not entirely satisfactory, prompting us to move further with the synthesis of the compound(s) using an adaptation of the procedure reported by Pozharskii, which is better described.

### Synthesis

After careful analysis of Pozharskii′s procedure, we determined that some minor adjustments could be made to prepare the described azo compound. At that time, the base n-butyllithium (*n*-BuLi) was generated in situ by reacting a butyl halide with lithium metal in an ethereal solvent, often a hydrocarbon−ether mixture. Nowadays, several lithium bases are available, and we decided to use one of the most common, *t*-butyllithium (*t*-BuLi). As for the solvent, and given the requirement of atmosphere oxidation, we decided to choose one with a relatively high boiling point, such as THF, to avoid fast evaporation (see Experimental Section for details). The treatment of 1*H*-indazol-3-amine **5** in dry THF with *t*-BuLi yields two compounds: a dark-red solid **6** and an orange-yellow solid **7**, in distinct yields depending on the equivalents of *t*-BuLi (Scheme 1). Analysis of their mass spectra and HRMS indicated that both have the same molecular formula, C_14_ H_10_ N_6_.

**Scheme 1:**
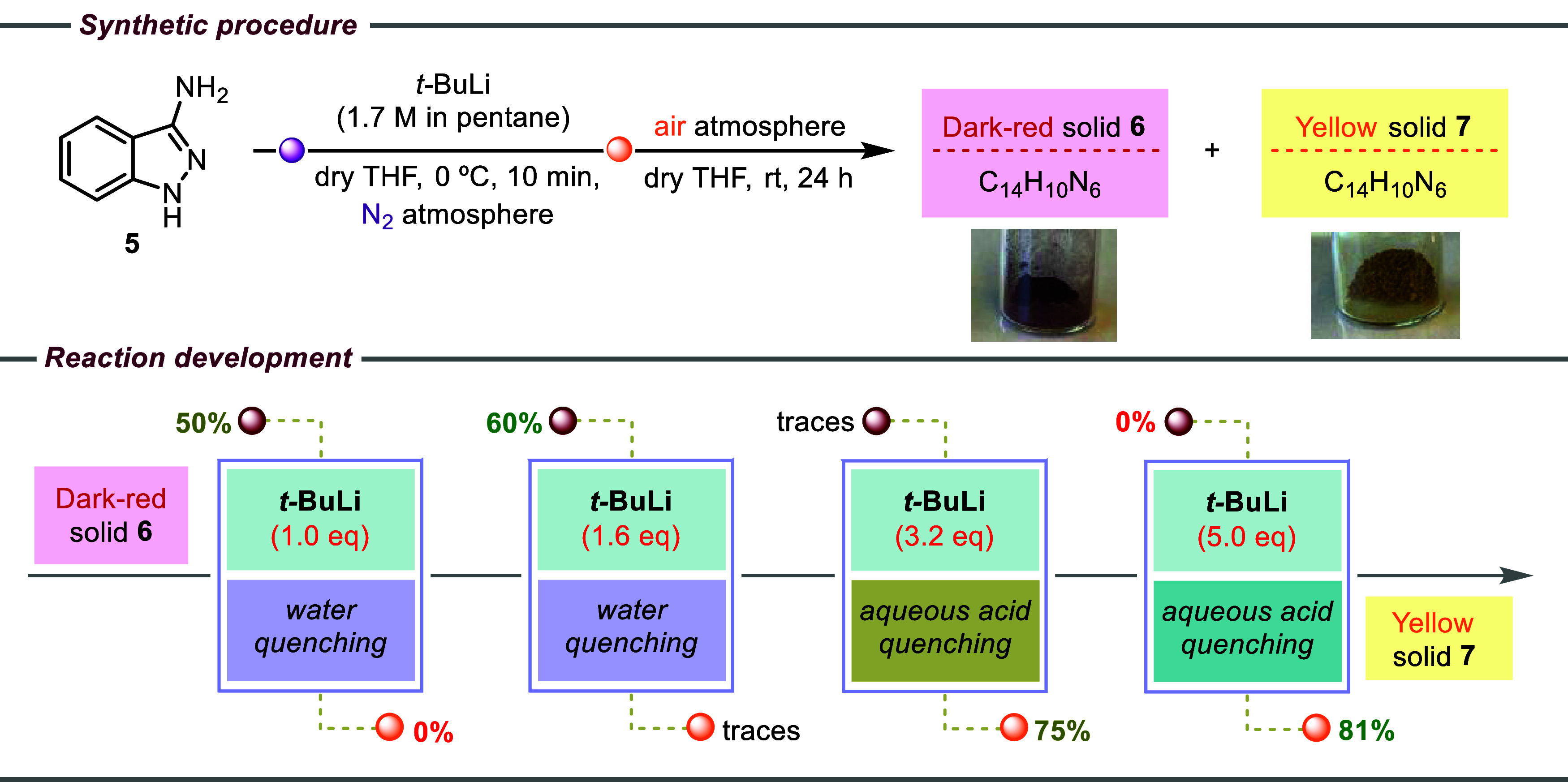
Oxidation of 1*H*-Indazol-3-amine **5** under Basic Conditions

When using 1.6 equiv of *t*-BuLi and performing a water workup of the reaction slurry, the red compound **6** is recovered upon filtration and purified by chromatography (60% isolated yield, see Experimental Section for details). The reduction of *t*-BuLi loading to 1.0 equiv gives compound **6** exclusively, however, with a slight decrease in the isolated yield from 60 to 50%. On the other hand, the use of 5 equiv of *t*-BuLi drives the reaction toward the exclusive synthesis of the yellow compound **7** in 81% isolated yield. An intermediate amount of *t*-BuLi (3.2 equiv) was attempted, delivering a similar yield, but with detectable traces of compound **6**. Both compounds were thoroughly characterized using a series of techniques, including UV–vis, FTIR, 1D/2D NMR, and X-ray diffraction, which will be further discussed in the subsequent sections. We began our analysis by using 1D/2D NMR techniques. The data from these techniques were also compared and discussed with the theoretical data.

### Structural Characterization

#### NMR and X-ray Diffraction

The information regarding the NMR spectra of both compounds is inconclusive: while the ^1^H NMR of the dark-red compound **6** points toward an unsymmetric structure (Figure S1), meaning that there are two different aromatic rings (Hünig), the ^1^H NMR of the yellow compound **7** (Figure S9) seems to correspond to a single aromatic ring (Pozharskii). In this setting, both the obtained compounds **6** and **7** were thoroughly analyzed using 1D and 2D NMR techniques (see SI for full details).

We began our analysis with the red-colored compound **6**. The first observation that puzzled us while analyzing the ^1^H NMR in DMSO-*d*_6_ was that we were dealing with an unsymmetrical compound, where two distinct spin systems are clearly identified (Figure 4A). The other interesting feature is the presence of an unusually deshielded signal at 9.34 ppm, which indicates that this proton might establish an intramolecular hydrogen bond. Additionally, the presence of one singlet at 8.02 ppm (2H integration), which does not correlate with any other proton, suggests that compound **6** probably still has a NH_2_ group, rather than two different NH groups as in the structures proposed by Hünig. Considering all the information: molecular formula of C_14_H_10_N_6_, two different aromatic rings, one NH_2_, and the structure of the starting material, we confidently propose the structure of compound **6**, which can assume an *E-* or Z-configuration (Figure 4A).

**Figure 4:**
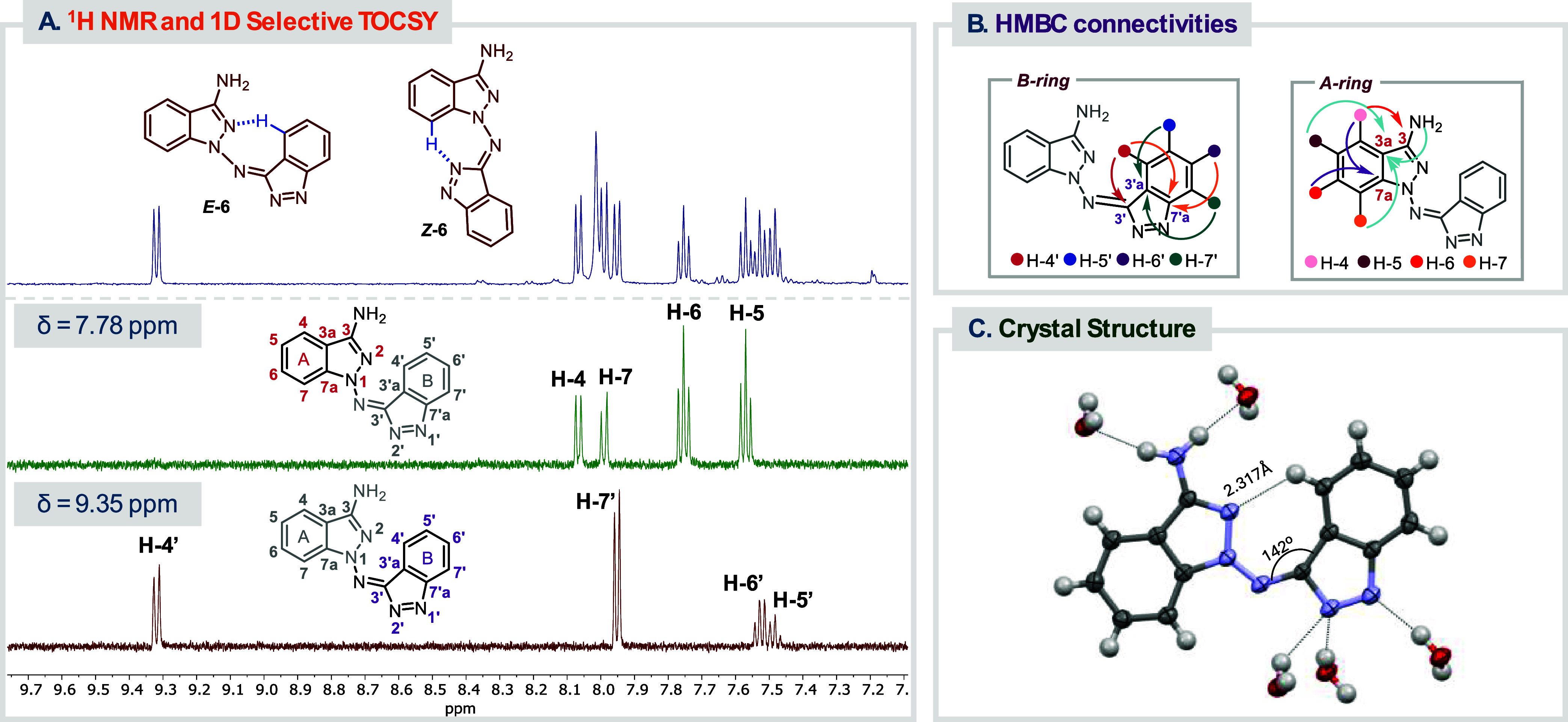
(A) ^1^H NMR (aromatic region) of compound **6** (top) and 1D Selective TOCSY irradiation at 7.78 ppm (middle), irradiation at 9.35 ppm (bottom) (500 MHz, DMSO-*d*_*6*_). (B) HMBC connectivities of A- and B-rings. (C) Single-crystal X-ray diffraction. Thermal ellipsoids are shown at the 50% probability level; hydrogen atoms are shown with an arbitrary radius (0.30 Å). C, gray; H, white; O, red; N, blue.

To unequivocally confirm that the molecule has two spin systems corresponding to rings A and B, 1D Selective TOCSY (Figure 4A) and 1D Selective Gradient TOCSY as a Function of Mixing Time (Figures S5–S6) were performed using two individual irradiations at 7.78 and 9.35 ppm, assuming the *E*-configuration of compound **6** (X-ray structure in Figure 3C, more detailed discussion in the X-ray diffraction section). Upon selective irradiation at 7.78 ppm (H-6), three correlation signals were observed at 8.09, 8.01, and 7.50 ppm. These signals correspond to the resonances of H-4, H-7, and H-5, respectively, indicating the A-ring spin system (Figure 4A, middle). In contrast, selective irradiation at 9.34 ppm (H-4′) produced correlation signals at 7.97, 7.55, and 7.50 ppm. These signals were assigned to the resonances of H-7′, H-6′, and H-5′, respectively, which belong to the B-ring system (Figure 4A, bottom).

Assuming the H-4′ as the most deshielded proton due to its intramolecular hydrogen bond with N-2, it is possible to assign the corresponding spin system of the B-ring. The HSQC spectrum of the compound enabled the assignment of hydrogen-linked carbons on the B-ring, while the HMBC spectrum allowed for the assignment of the remaining unprotonated carbons C-3′a (119.1 ppm), C-7′a (158.7 ppm), and C-3′ (153.4 ppm) (Figure 4B). The same approach was used to assign carbons C-3a (120.9 ppm), C-7a (142.4 ppm), and C-3 (157.2 ppm). Clear HMBC correlations of H-5, H-7, and NH_2_ with C-3a are observed, with the latter being crucial to distinguish between C-3a and C-3′a (Figure 4B). The red compound **6** crystallized with two water molecules (Figure 4C), involved in a network of hydrogen bonds with the amino and indazo groups. The bond lengths confirm the single and double bonds localization, and the *E*-configuration of the C=N double bond between the two indazo rings. Surprisingly, the two indazo rings are coplanar (dihedral angle 178°–179°), with a strong intramolecular hydrogen bond (distance N···H–C of 2.317 Å): this strong hydrogen bond is supported by the NMR analysis, with a signal at 9.34 ppm for the proton H-4′. In order to accommodate this hydrogen bond and the coplanarity of the aromatic rings, the bond angles have to distort dramatically: in the seven-membered ring formed, the C–C=N angle increases to 142°, compared to the usual 120°.

In turn, when analyzing the ^1^H NMR of the orange-yellow compound **7** in DMSO-*d*_6_ (Figure 5A), we noticed that the two spin systems were no longer observable, clearly contrasting with those of compound **6**. The 1D selective TOCSY confirms the existence of only one spin system, suggesting that compound **7** is symmetric (see SI for full details). As in the case of compound **6**, the signal of H-4 (8.36 ppm) is relatively deshielded, probably because of its interaction with an N atom of the diazo moiety. A broad singlet is also observed at 13.88 ppm and assigned to the NH proton of the indazole rings (Figure 5A). The HMBC shows connectivities between the NH signal and C-3 (156.4 ppm) and C-7a (142.1 ppm) signals, allowing us to confirm that the broad singlet at 13.88 ppm belongs to the NH group of indazole rather than an NH_2_, as observed in compound **6** (Figure 5B).

**Figure 5:**
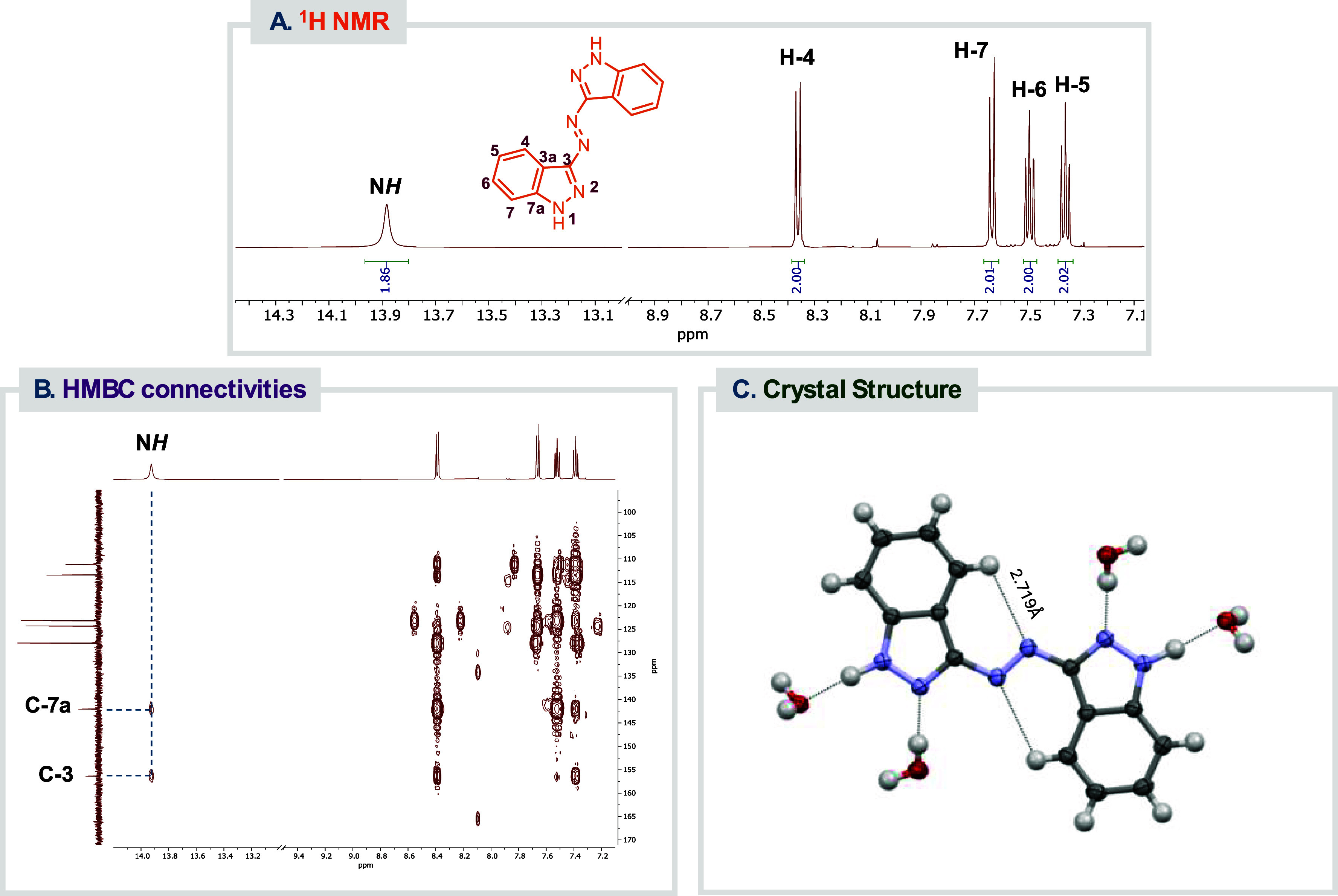
(A) ^1^H NMR (aromatic region) of compound **7** (500 MHz, DMSO-*d*_6_). (B) HMBC connectivities. (C) Single-crystal X-ray diffraction. Thermal ellipsoids are shown at the 50% probability level; hydrogen atoms are shown with an arbitrary radius (0.30 Å). C, gray; H, white; O, red; N, blue.

The yellow-orange compound **7** crystallized with one water molecule (Figure 5C, the asymmetric unit is half a molecule and half a water), which is involved in a network of hydrogen bonds holding the molecules together. The bond lengths also confirm the localization of the single and double bonds and therefore confirm the structure which corresponds to the structure proposed after NMR analysis: the azo group presents an *E*-configuration, and the NH group is on the indazo group without tautomerization. The proton H-4 is hydrogen bonded with the azo group, which is in accordance with the NMR analysis, this proton appearing around 8.4 ppm.

Table 1 compares the experimental and calculated chemical shifts referring to red compound **6** and the yellow compound **7**. The protons of the amino group are very dependent on the solvent; in DMSO-*d*_6_, they are moved downfield. From the 37 chemical shifts of Table 1, we have calculated the simple linear equations with and without the ^15^N data

$$\mathrm{Exp}.=\left(3.4\pm 0.9\right)+\left(0.971\pm 0.008\right)\mathrm{calc}.,n=37,R^{2}=0.998$$

$$\mathrm{Exp}.=\left(0.6\pm 0.7\right)+\left(1.004\pm 0.007\right)\mathrm{calc}.,n=34,R^{2}=0.998$$

**Table 1:**
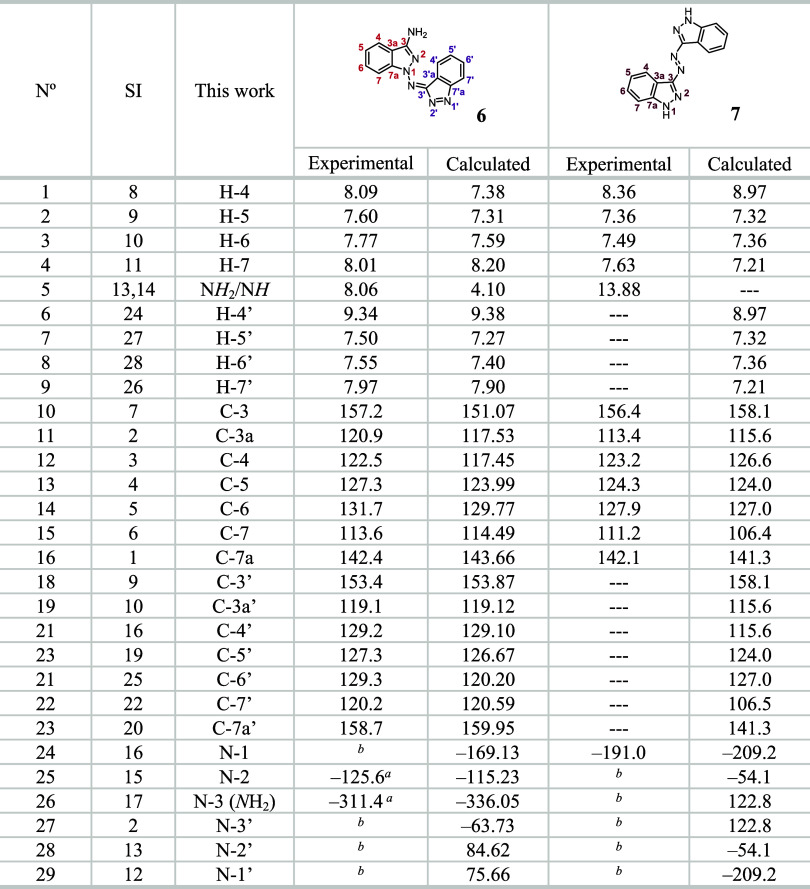
Comparison between Experimental and Calculated Chemical Shifts (in ppm) of Compounds **6** and **7**

a Measured from urea at +76.36 ppm. Corrected chemical shifts (from NH_3_ to nitromethane).

b Not observed.

In Table 2, there is a comparison between the calculated ^15^N chemical shifts for the three previously reported structures **1** (identical to **7**), **2**, and **3**, as well as our new compound **6**. Obviously, ^15^N chemical shifts, if complete, will allow us to establish the structure of the four isomers.

**Table 2:** ^15^N NMR Signals from More Positive Downwards^a^

	1 ≡ 7	2	3	6
N-1	–209.2	–225.0	–224.3	–169.1
N-2	–54.1	–116.3	–102.0	–115.2
N-3 (NH_2_)	122.8	46.8	–224.0	–336.0
N-3′	122.8	76.1	–64.3	–63.7
N-2′	–54.1	–88.1	20.3	84.6
N-1′	–209.2	–174.3	71.9	75.7

a Only the N atom linked to H atoms is observed.

#### Melting Point Comparison

After the elemental analysis, the main characterization reported by Hünig and Pozharskii is the melting point. The dark-red compound **6** melts at 239–241 °C, and the yellow compound **7** melts at 298–300 °C. Hünig does not report a melting point (mp) but sends to Bamberger and Wildi’s 1906 paper, where they reported a mp of 228 °C.^20^ Pozharskii obtained a yellow solid, which crystallized as orange crystals melting at 330 °C (decomposition, crystallized in ethanol-dimethylformamide). These results indicate that the dark-red compound **6** is likely the compound obtained by Hünig, and compound **7** is similar to the one obtained by Pozharskii. Melting points are extremely difficult to predict or model, and therefore theoretical modelization is not useful at this step.

#### UV–Vis Spectra of Compounds **6** and **7**

The second characterization reported by Hünig and Pozharskii is the color of the obtained compounds, and accordingly, we have calculated theoretical UV–vis spectra using TD-DFT at the PBE0 level in gas phase and EtOH and CH_3_CN (Figure 6) and acquired experimental data regarding the absorbance spectra of the compounds we obtained (Figure 6). The maximum absorption bands were identified at 395, 438, and 438 nm, in gas phase, EtOH, and CH_3_CN, respectively (Figure 6A). The UV–vis spectra of compound **6** (Figure 6A) and compound **7** (Figure 6B) were acquired in EtOH and CH_3_CN at 0.01 μM, respectively. In EtOH, compound **6** shows a maximum absorption band at 503 nm, while in CH_3_CN the absorption band shifts to 479 nm (Figure 6A). Regarding compound **7**, the UV–vis shows an absorption band at 383 nm in EtOH, shifting to 376 nm in CH_3_CN (Figure 6B). The UV band of compound **7** resembles the data reported by Pozharskii et al. of a UV band at 390 in MeOH (Δν = 7 nm). Hünig reported an absorption maximum of the deep-red compound (**4**H_Red_) at 505 nm in CH_3_CN, while the yellow **4**_Red_ absorbs at 395 nm. Our data concerning the dark-red compound **6** is closer to that reported by Hünig for the deep-red compound (**4**H_Red_) at 505 nm (Δν = 26 nm). This suggests that the deep-red compound (**4**H_Red_) should have the structure of our compound **6**.

**Figure 6:**
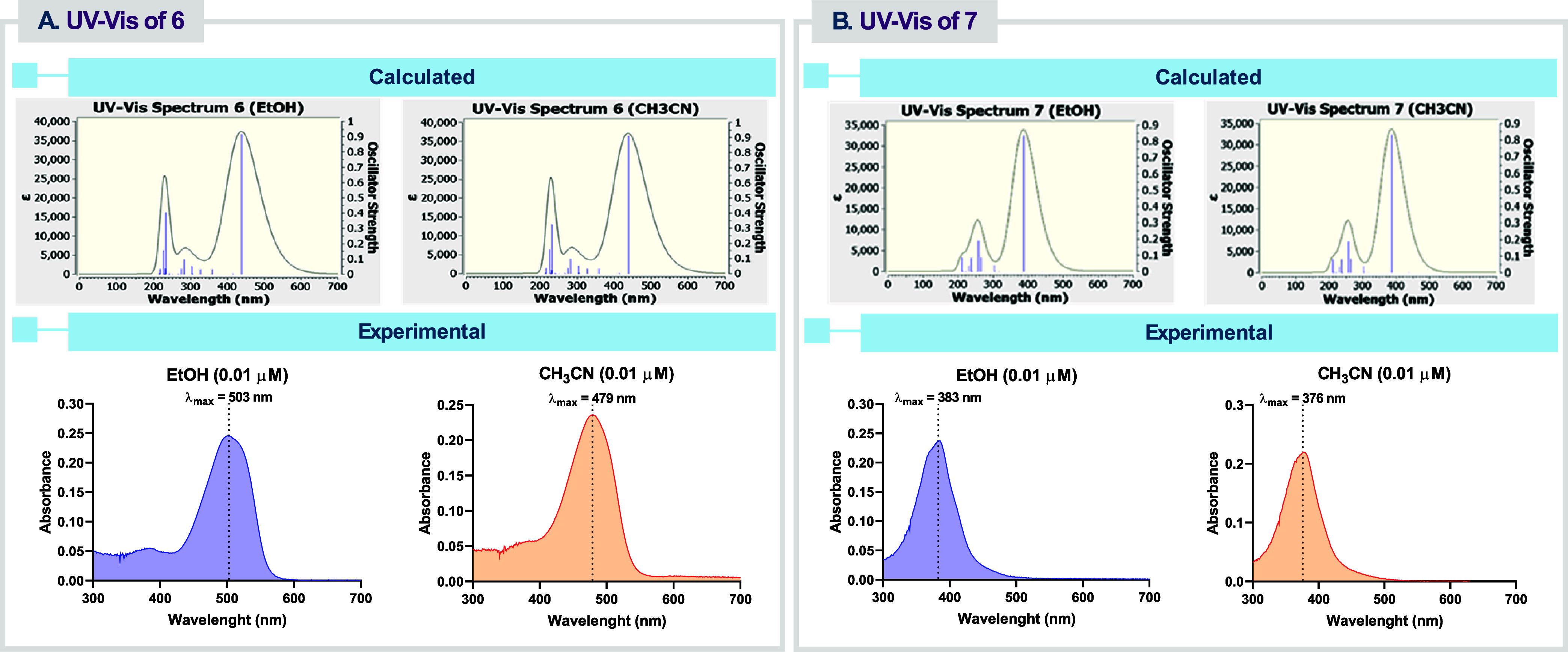
(A) UV–vis of compound **6** calculated in gas phase (top), experimental (bottom), in EtOH (left) and CH_3_CN (right). (B) UV–vis of compound **7** calculated in gas phase (top), experimental (bottom), in EtOH (left), and CH_3_CN (right).

#### FTIR Spectra

Next, we moved to FTIR data, calculating them and scaling by a factor of 0.9613 corresponding to B3LYP/6–311+G­(d,p) calculations.^21^ Two stretching NH bands, the symmetric and the antisymmetric, were calculated for compound **6** at 3582 (s) and 3689 (as) cm^–1^. The difference calculated, gas phase —experimental (KBr), average values for the two bands, is Δν = 436 cm^–1^. We have also acquired ATR-FTIR and spectra of compounds **6** and **7** (Figure 7). The experimental values determined in the solid state are systematically shifted from our calculations. The FTIR spectrum of the compound **6** (Figure 7A) shows stretching bands at ∼3100 and ∼3300 cm^–1^, very similar to those presented by compound **7** (Figure 7B).

**Figure 7:**
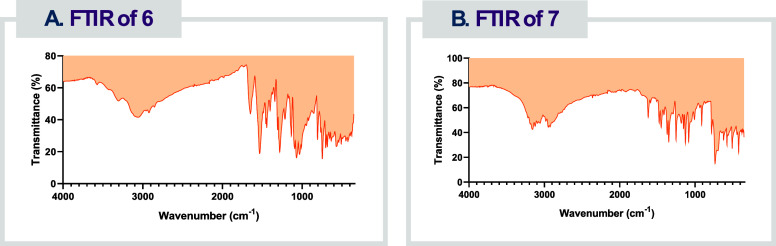
FTIR spectrum of compound **6** (left) and compound **7** (right).

#### Proposed Mechanism

Having established that in basic conditions the compounds **6** and **7** can be formed, it remains to explain why constitutional isomer **6** is preferably formed when 1.6 equiv of *t*-BuLi are used, despite isomer **7** being thermodynamically more stable by 55 kJ·mol^–1^ (Scheme 2). In a very simplified manner, we propose that this nonexpected result originates from the deprotonation steps preceding oxidation by air, which occur under the experimental conditions employed by both Hünig^14^ and Pozharskii.^19^ Notably, one of the most widely used methods for the synthesis of azo compounds is the Baeyer–Mills reaction (first published by Baeyer in 1874 and further investigated by Mills). This reaction involves the condensation of anilines and nitroso compounds and can be performed either in acidic or basic conditions. Typically, the nitroso arene component is generated in situ by either oxidation of an aniline or reduction of a nitrobenzene.

**Scheme 2:**
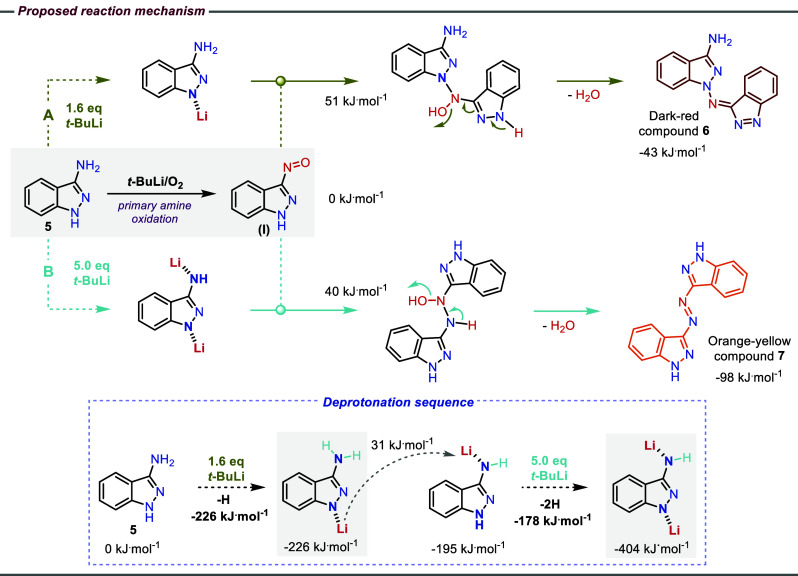
Proposed Mechanism for the Synthesis of Dark-Red Compound **6** and Orange-Yellow Compound **7**

In the case of 1*H*-indazol-3-amine **5**, it is conceivable that it undergoes a stepwise deprotonation sequence with increasing equivalents of *t*-BuLi, which significantly influences the reaction outcome (Scheme 2, inset). A pivotal step may be the initial deprotonation, which should preferentially occur at the indazole NH, alongside concomitant deprotonation in the primary amine (at least in a small extension) (Scheme 2, A pathway). This mechanistic event promotes the subsequent Baeyer–Mills reaction toward compound **6**. To provide a qualitative rationale for experimental regioselectivity, computational calculations were performed using a simplified model. While this approach does not explicitly account for the lithium coordination sphere, solvation by THF, or potential aggregation factors known to influence absolute energetic values, it supported experimental observations. The first deprotonation of indazole NH was theorized to be 31 kJ mol^–1^ more favorable relative to the primary amine (Scheme 2, inset), which may help to rationalize the predominance of compound **6** at lower base loads. An indication of a more nucleophilic indazole is supported by the NBO charge of indazole (−0.370) becoming more negative (−0.579) at this deprotonation step. By increasing the *t*-BuLi load, deprotonation of both indazole NH and the primary amine becomes more likely, therefore shifting the reaction toward compound **7** (Scheme 2, B pathway). At higher base loads, with support by NBO charge, the higher intrinsic nucleophilicity of the primary amine recovers the gap to imidazole nitrogen, which contributes to once again favoring compound **7** during the deprotonation sequence of **5**. A common feature of both mechanistic pathways A and B is the formation of nitroso intermediate **I** upon oxidation of the primary amine of compound **5** in basic conditions (Scheme 2), which subsequently reacts with nucleophilic intermediates to yield compounds **6** or **7**.

This analysis indicates that the reaction reported by Hünig most likely produced a mixture of compounds **6** and **7** rather than a single constitutional isomer. Both authors reported colored compounds from orange to deep red, which is consistent with the colors observed for compounds **6** and **7**. The proportion of each compound is likely to vary with the basicity degree of the reaction media and the selected solvent. The presence of two nucleophilic sites in the starting 1*H*-indazol-3-amine **5** is the key factor that enables divergent mechanistic pathways, making this reaction significantly more sensitive than previously recognized and accessed. The inherently higher nucleophilicity of primary amine is counterbalanced by the formation of a more stable intermediary arising from indazole N–H deprotonation at low base loadings, allowing the on-demand control of the reaction.

## Conclusions

In this communication, we investigated the products obtained from the oxidation in basic conditions of 1*H*-indazol-3-amine. We found that the reaction outcomes are dependent on the amount of base employed in the reaction. According to Hünig′s report, the reaction produces a deep-red compound (named as **4**H_Red_) absorbing at 505 nm in acetonitrile. Very scarce additional information is given, but Hünig mentioned at a certain point that there is no symmetry of the splitting signals in the ^1^H NMR. According to our data, the absence of symmetry in the spin systems can be attributed to the unsymmetric structure of our dark-red compound **6**, which we fully characterized in this study for the first time. We also verify that upon excess of *t*-BuLi, the oxidation of 1*H*-indazol-3-amine produces the diazene compound **7**, which, unlike the Hünig′s report, indeed has symmetry. In summary, the oxidation of 1*H*-indazol-3-amine in basic conditions most likely delivers two structures, depending on the amount of base: the (*E*)-1-[(3*H*-indazo-3-ylidene)­amino]-1*H*-3-amine **6**, reported here for the first time, and a symmetric compound **7**. Both compounds are synthesized through Mills reaction, but upon distinct nucleophilic attack, from the primary amine or by the nucleophile generated from proton abstraction in the NH group of the indazole.

## Experimental Section

### Synthesis of (*E*)-1-[(3*H*-Indazo-3-Ylidene)­Amino]-1*H*-3-Amine (6)

1*H*-Indazol-3-amine **5** (1.5 mmol, 200 mg) was dissolved in dry THF (8 mL) at room temperature under a nitrogen (N_2_) atmosphere. The solution was then cooled to 0 °C using an ice bath. *t*-Butyllithium (*t*-BuLi, 1.3 mL, 2.25 mmol, 1.7 M solution in pentane) was added dropwise, and the reaction mixture was stirred for 10 min at 0 °C. The reaction vessel was subsequently opened, allowing the mixture to oxidize under an air atmosphere for 24 h. After this period, 10 mL of distilled water was added, and the mixture was stirred for an additional 10 min until a brown precipitate formed. The precipitate was removed by filtration and rinsed with cooled hexane (0 °C, 10 mL). Following purification by column chromatography using a gradient of 0% to 100% ethyl acetate in hexane, the (*E*)-1-[(3*H*-indazo-3-ylidene)­amino]-1*H*-3-amine **6** was obtained.

**Caution:** *t*-Butyllithium is extremely pyrophoric. It must be handled using proper needles and syringe techniques. All manipulations were performed on the smallest practical scale.

#### (*E*)-1-[(3*H*-Indazo-3-Ylidene)­Amino]-1*H*-3-Amine (**6**)

Dark-red solid, 118 mg, 60% yield; mp 239–241 °C. **^1^H NMR (DMSO-*d*_6_, 500 MHz)**: δ = 9.34 (dd, 1H, H-4′, *J* = 7.3, 1.3 Hz), 8.09 (dd, 1H, H-4, *J* = 7.9, 1.0 Hz), 8.06 (s, 2H, 3-NH_2_), 8.01 (dd, 1H, H-7, 7.2, 0.9 Hz), 7.97 (dd, 1H, H-7′, *J* = 7.6, 1.2 Hz), 7.77 (ddd, 1H, H-6, *J* = 8.1, 7.2, 0.9 Hz), 7.59 (ddd, 1H, H-5, *J* = 8.1, 7.9, 0.9 Hz), 7.55 (ddd, 1H, H-6′, *J* = 7.6, 7.4, 1.3 Hz), 7.50 (ddd, 1H, H-5′, *J* = 7.4, 7.3, 1.2 Hz) ppm. **^13^C­{^1^H} NMR (DMSO-*d*_6_, 126 MHz)**: δ = 158.7 (C-7′a), 157.2 (C-3), 153.4 (C-3′), 142.4 (C-7a), 131.7 (C-6), 129.3 (C-6′), 129.2 (C-4′), 128.4 (C-5′), 127.3 (C-5), 122.5 (C-4), 120.9 (C-3a), 120.2 (C-7′), 119.1 (C-3′a), 113.6 (C-7) ppm. **^15^N NMR (DMSO-*d*_6_, 51 MHz)**: δ = 70.28 (NH_2_), 256.15 (N-2) ppm. **HRMS (ESI)**: *m*/*z*: [M + H]^+^ calcd for C_14_H_11_N_6_, 263.1040; found, 263.1051.

### Synthesis of (*E*)-1,2-di­(1*H*-Indazol-3-yl)­Diazene (**7**)

1*H*-Indazol-3-amine **5** (1.5 mmol, 200 mg) was dissolved in dry THF (8 mL) at room temperature under a nitrogen (N_2_) atmosphere. The solution was then cooled to 0 °C using an ice bath. *t*-Butyllithium (*t*-BuLi, 4.4 mL, 7.5 mmol, 1.7 M solution in pentane) was added dropwise, and the reaction mixture was stirred for 10 min at 0 °C. The reaction vessel was subsequently opened, allowing the mixture to oxidize under an air atmosphere for 16 h (TLC monitoring). After this period, the reaction mixture was quenched with water (10 mL) and the pH adjusted to 4 with HCl (10%). The dark-yellow precipitate was then recovered by filtration, giving the (*E*)-1,2-di­(1H-indazol-3-yl)­diazene **7** in its pure form.

**Caution:** *t*-Butyllithium is extremely pyrophoric. It must be handled using proper needle and syringe techniques, and all manipulations were performed on the smallest practical scale.

#### (*E*)-1,2-Di­(1*H*-Indazol-3-yl)­Diazene (**7**)

Yellow solid, 160 mg, 81% yield; mp 298–300 °C. **^1^H NMR (DMSO-*d*_6_, 500 MHz)**: δ = 13.88 (br s, 2H, NH), 8.36 (dd, 2H, H-4, *J* = 8.0, 1.0 Hz), 7.63 (dd, 2H, H-7, *J* = 8.3, 1.0 Hz), 7.49 (ddd, 2H, H-6, *J* = 8.3, 6.9, 1.0 Hz), 7.36 (ddd, 2H, H-5, *J* = 8.0, 6.9, 1.0 Hz) ppm. **^13^C­{^1^H} NMR (DMSO-*d*_6_, 126 MHz)**: δ = 156.4 (C-3), 142.0 (C-7a), 127.9 (C-6), 124.3 (C-5), 123.2 (C-4), 113.4 (C-3a), 111.2 (C-7) ppm. **^15^N NMR (DMSO-*d*_6_, 51 MHz)**: δ = 190.71 (N-1) ppm. **HRMS (ESI)**: *m*/*z*: [M + H]^+^ calcd for C_14_H_11_N_6_, 263.1040; found, 263.1042.

### DFT Calculations

The geometry of the molecules was optimized with the B3LYP functional^22,23^ and the 6–311++G­(d,p) basis set.^24^ The empirical dispersion D3 with the Becke–Johnson damping,^25,26^ D3­(BJ), has been included in the calculations. Frequency calculations have been carried out at the same computational level to confirm that the structures correspond to energetic minima. The solvent has been modeled with the PCM approximation^27^ and the standard parameters for EtOH and CH_3_CN. The NMR chemical shifts have been calculated with the GIAO approximation,^28^ and the B3LYP/6–311++G­(d,p) computational level. TD-DFT calculations at the PBE0/6–311++G­(d,p) level^29^ have been used to predict the UV spectra. All of the calculations have been carried out with the Gaussian-16 program.^30^

## Supplementary Material



## Data Availability

The data underlying this study are available in the published article and its Supporting Information.

## Notes

^1^H, ^13^C, ^1^H–^13^C HSQC, ^1^H–^13^C HMBC, ^1^H–^15^N HSQC, ^1^H–^15^N HMBC spectra for all new products, X-ray diffraction of compounds **6** and **7**, and mechanistic analysis of deprotonation sequence (PDF)
