# Supplementary material for: The Structures Obtained from the Oxidation of 3‑Amino‑1H‑indazole in Basic Conditions According to Hünig and Pozharskii
Source: J Org Chem. 2026 Jun 17;91(26):8784–93. doi: 10.1021/acs.joc.6c00198 (PMC13339642; doi:10.1021/acs.joc.6c00198)
Supplement: Supplementary file 1 [file jo6c00198_si_001.pdf]

Supporting Information

The structures obtained from the oxidation of 3-amino-1*H*-indazole in basic conditions according to Hünig and Pozharskii

Hélio M. T. Albuquerque,[a] Luís F. B. Fontes,[a,b] Samuel Guieu,[a] Artur M. S. Silva,*[,a] Ibon Alkorta*[,c] and José Elguero[c]

[a]*LAQV-REQUIMTE & Department of Chemistry, Universidade de Aveiro, Aveiro 3810-193, Portugal.*

[b]*CICECO & Department of Chemistry, Universidade de Aveiro, Aveiro 3810-193, Portugal.*

[c]*Instituto de Química Médica, CSIC, Juan de la Cierva, 3, E-28006 Madrid, Spain.*

Index

S3 **General Information**

S4 **NMR spectra of compound 6**

S4 **Figure S1.** ^{1}H-NMR of compound **6** (500 MHz, DMSO-d_6)

S4 **Figure S2.** ^{13}C-NMR of compound **6** (126 MHz, DMSO-d_6)

S5 **Figure S3.** ^{1}H-^{13}C HSQC of compound **6** (500 MHz, DMSO-d_6)

S5 **Figure S4.** ^{1}H-^{13}C HMBC of compound **6** (500 MHz, DMSO-d_6)

S6 **Figure S5.** Selective TOCSY of compound **6** upon irradiation at δ = 9.346 ppm and varying the mixing time (500 MHz, DMSO-d_6).

S6 **Figure S6.** Selective TOCSY of compound **6** upon irradiation at δ = 7.781 ppm and varying the mixing time (500 MHz, DMSO-d^6).

S7 **Figure S7**. ^{1}H-^{15}N HSQC of compound **6** (500 MHz, DMSO-d_6).

S7 **Figure S8.** ^{1}H-^{15}N HMBC of compound **6** and HMBC projection (inset) (500 MHz, DMSO-d_6).

S8 **NMR spectra of compound 7**

S8 **Figure S9.** ^{1}H-NMR of compound **7** (500 MHz, DMSO-d_6).

S8 **Figure S10.** ^{13}C-NMR of compound **7** (126 MHz, DMSO-d_6)

S9 **Figure S11.** ^{1}H-^{13}C HSQC of compound **7** (500 MHz, DMSO-d_6)

S9 **Figure S12.** ^{1}H-^{13}C HMBC of compound **7** (500 MHz, DMSO-d_6)

S10 **Figure S13.** ^1H-^{15}N HSQC of compound **7** and HSQC projection (inset) (500 MHz, DMSO-d_6**)**

S11 **X-ray diffraction of compounds 6 and 7**

S14 **Mechanistic analysis of deprotonation sequence**

S15 **Figure S14**. Global mechanistic view of isomer **6** (red) and **7** (yellow) pathway considering compound **5** and nitrosoarene (grey) as starting point (0 kJ.mol^{-1}) with product values including H_2O molecule.

S15 **Figure S15**. First deprotonation step of compound **5** reaction with *t*-BuLi tracking primary amine (yellow) or imidazole (red) to yield isomer **6** and **7** single deprotonation intermediates and *t*-Butyl.

S16 **Figure S1.** HOMO and localized NBO charge (in Hartree) progression of primary amine (blue) and pyrazole (green) during deprotonation sequence with *t*-BuLi. Highlight (red) for the lowest and higher NBO charge gaps between primary amine and pyrazole amine.

S16 **Figure S2.** Localized NBO charges of each intermediate in the deprotonation sequence as an indication of nucleophilicity progression.

S17 **Table S1.** Electronic energy (Hartree), number of imaginary frequencies and cartesian coordinates (Å) of **6** and **7** at B3LYP/6-311++G(d,p)/D3(BJ) computational level.

General Information

Unless otherwise stated, all glassware was oven-dried before use and the reactions were performed under nitrogen (N_2) atmosphere. Solvents of commercial grade were used as received unless otherwise stated. All reagents were used as received from commercial suppliers unless otherwise stated.

Reaction progress was monitored by thin-layer chromatography (TLC) performed on aluminum plates coated with silica gel F254 with 0.2 mm thickness. TLC chromatograms were visualized by fluorescence quenching with UV irradiation at 254 nm (or 366 nm). Flash column chromatography was performed using a Teledyne ISCO Combiflash® RF 100 with a Teledyne ISCO RediSep® Silica Gel Flash Column (40 grams).

NMR spectra were recorded with a Bruker Avance III TM HD - 500 spectrometer with CryoProbe Prodigy BBO (500 MHz for ^{1}H, 126 MHz for ^{13}C and 51 MHZ for ^{15}N). Chemical shifts (δ) are reported in ppm and coupling constants (*J*) in Hz; the internal standard was tetramethylsilane (TMS). Unequivocal ^{13}C assignments were made with the aid of 2D gHSQC and gHMBC (delays for one-bond and long-range *J* C/H couplings were optimised for 145 and 7 Hz, respectively) experiments. ^{1}H or ^{13}C NMR splitting patterns were designated as singlet (s), doublet (d), triplet (t), quartet (q), pentet (p). Splitting patterns that could not be interpreted or easily visualized were designated as multiplet (m) or broad (br).

Melting points were measured in a Büchi B-540 apparatus fitted with a microscope and are uncorrected.

High resolution mass spectra analysis (HRMS-ESI) was performed on a microTOF (focus) mass spectrometer. Ions were generated using an ApolloII (ESI) source. Ionization was achieved by electrospray, using a voltage of 4500 V applied to the needle, and a counter voltage between 100 and 150 V applied to the capillary.

NMR spectra of compound 6

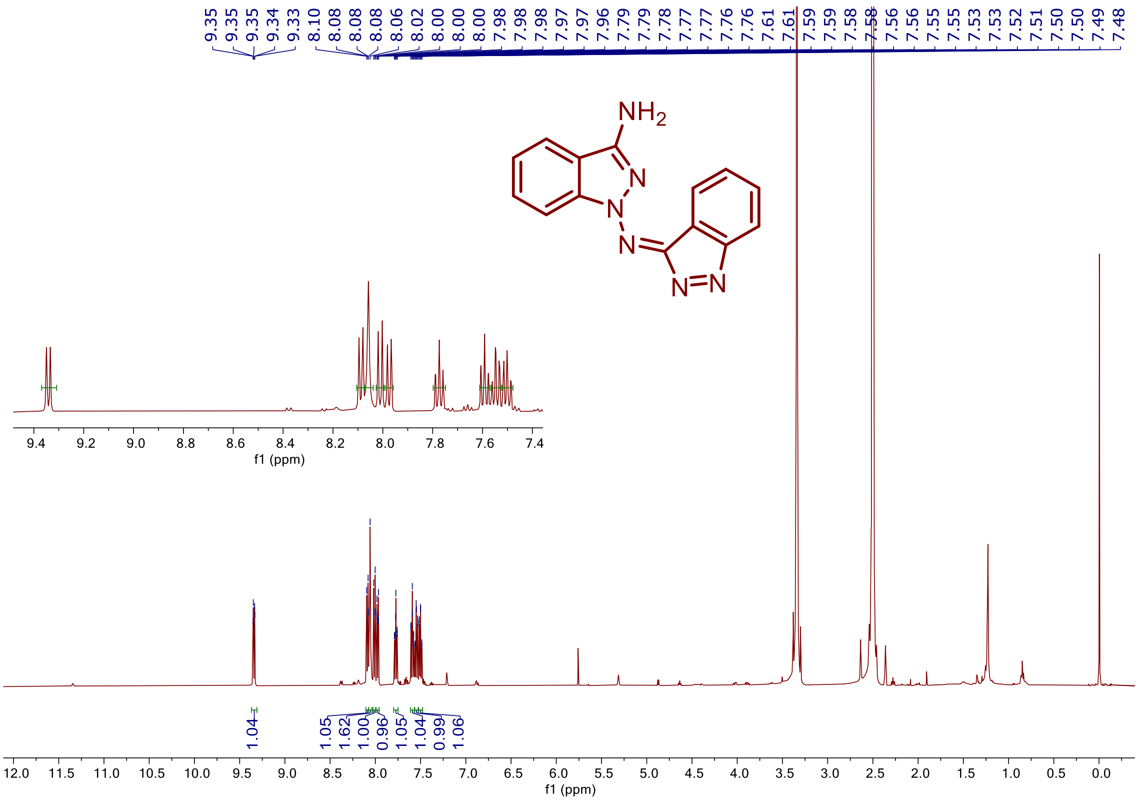

Figure S3. ^{1}H NMR of compound **6** (500 MHz, DMSO-d_6).

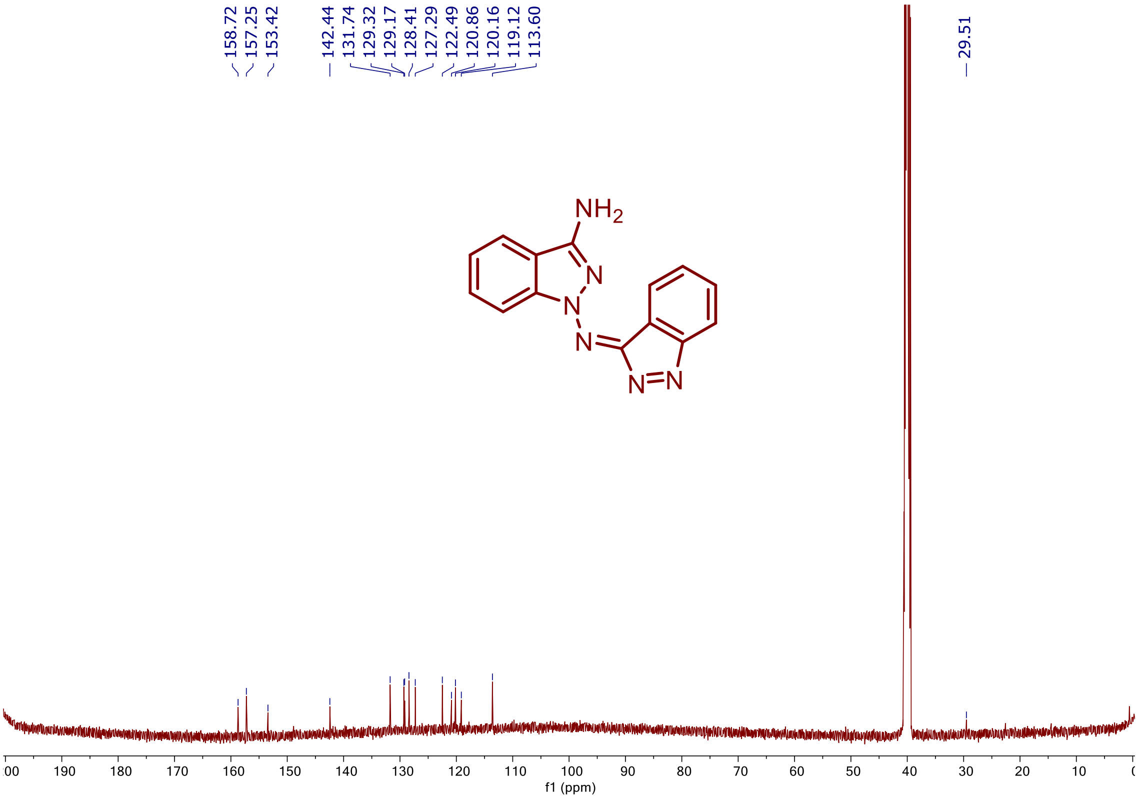

Figure S4. $^{13}C\{^{1}H\}$ NMR of compound **6** (126 MHz, DMSO-d_6).

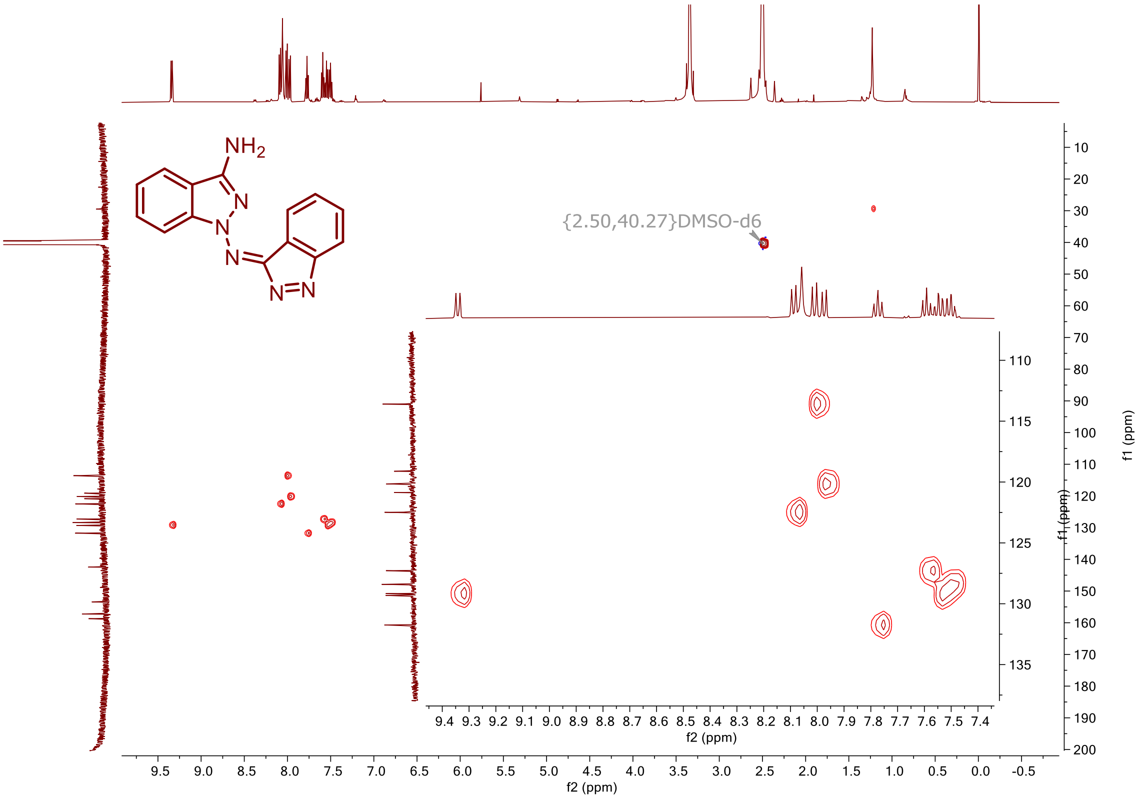

Figure S5. ^{1}H-^{13}C HSQC of compound **6** (500 MHz, DMSO-d_6).

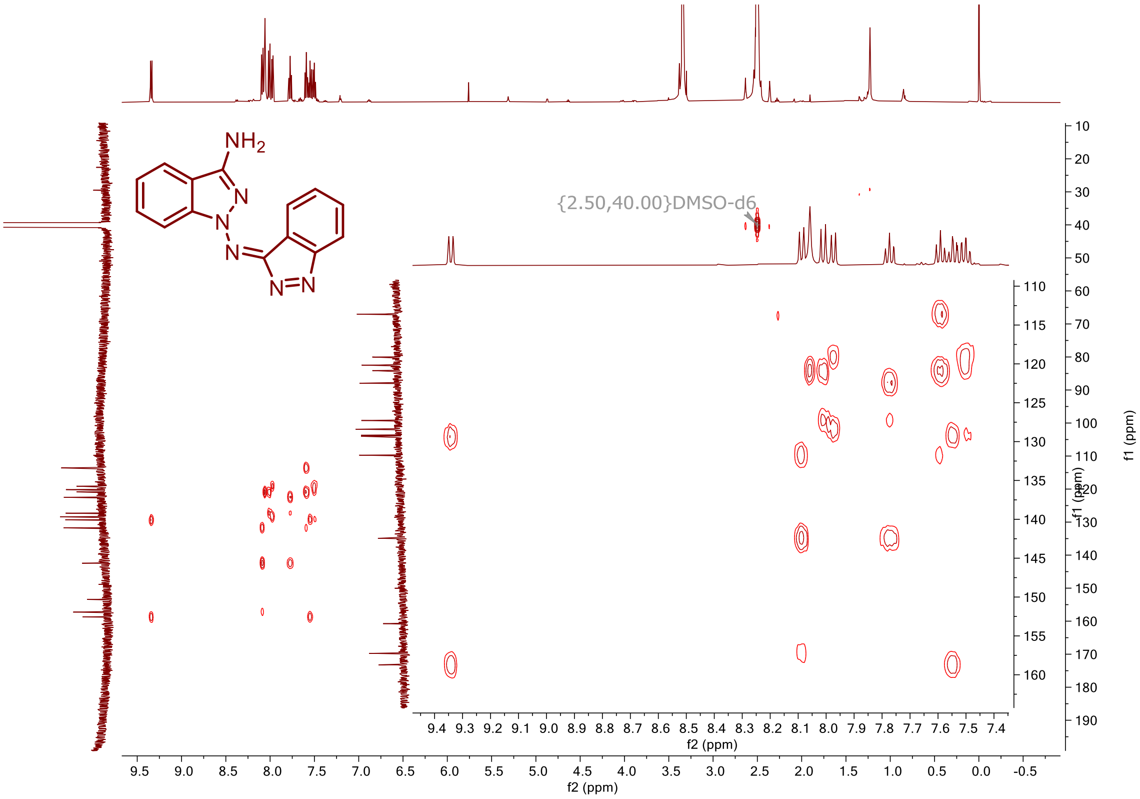

Figure S6. ^{1}H-^{13}C HMBC of compound **6** (500 MHz, DMSO-d_6).

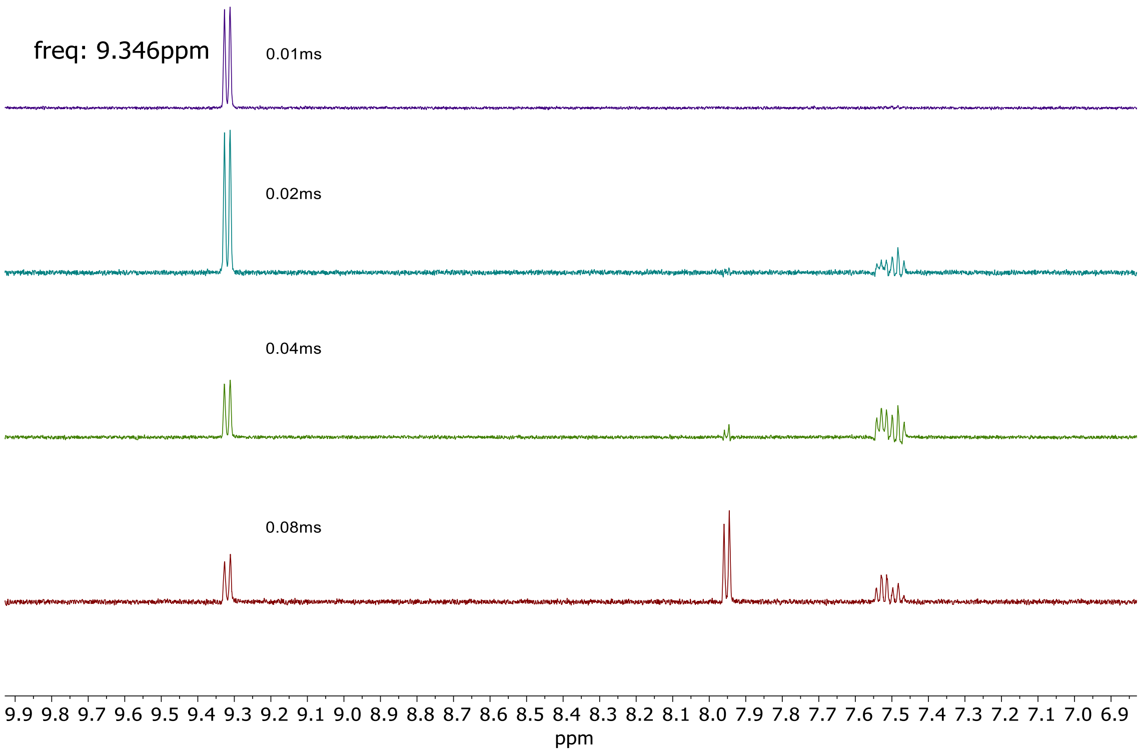

Figure S7. Selective TOCSY of compound **6** upon irradiation at **δ = 9.346** ppm and varying the mixing time (500 MHz, DMSO-*d*$_6$).

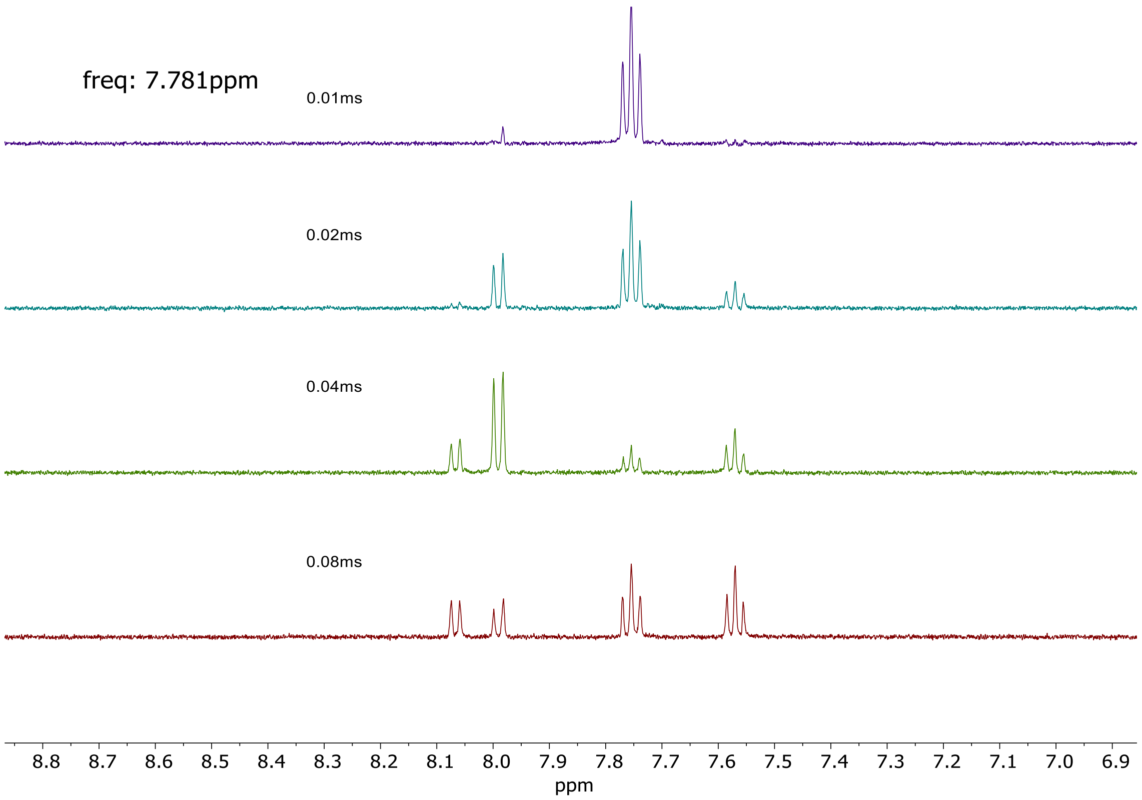

Figure S8. Selective TOCSY of compound **6** upon irradiation at **δ = 7.781** ppm and varying the mixing time (500 MHz, DMSO-*d*$_6$).

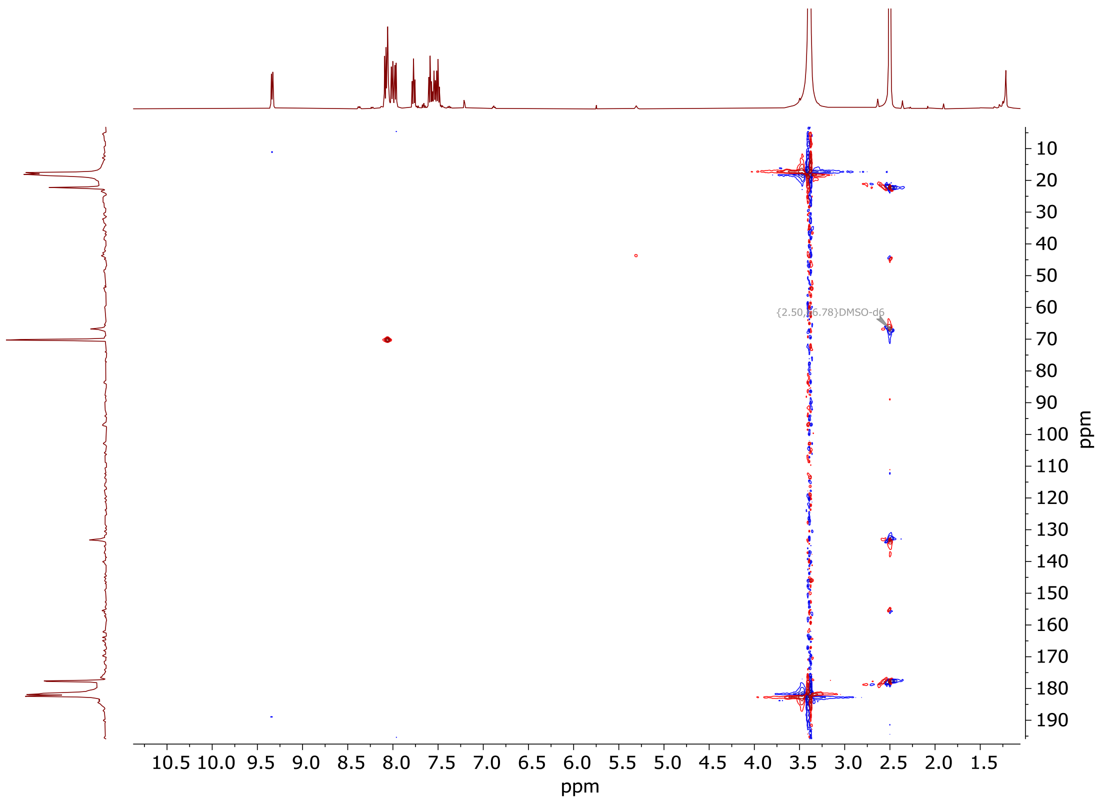

Figure S9. ^{1}H-^{15}N HSQC of compound **6** (500 MHz, DMSO-d_6).

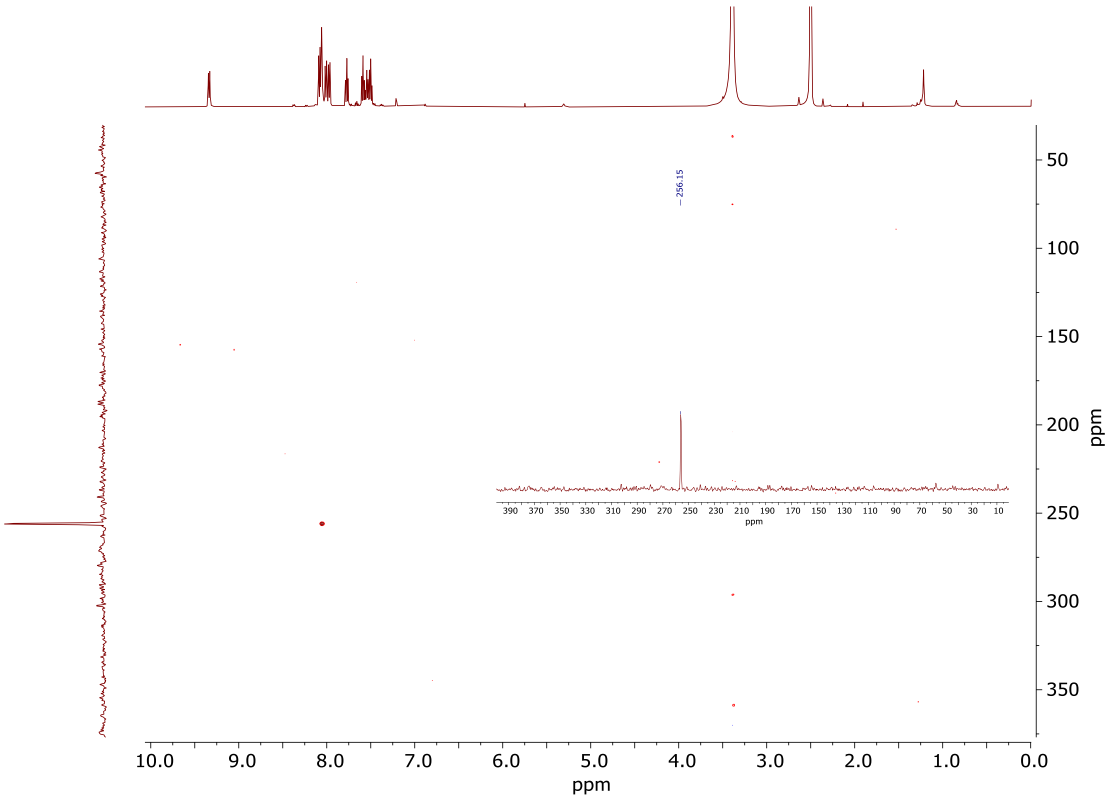

Figure S10. ^{1}H-^{15}N HMBC of compound **6** and HMBC projection (inset) (500 MHz, DMSO-d_6).

NMR spectra of compound 7

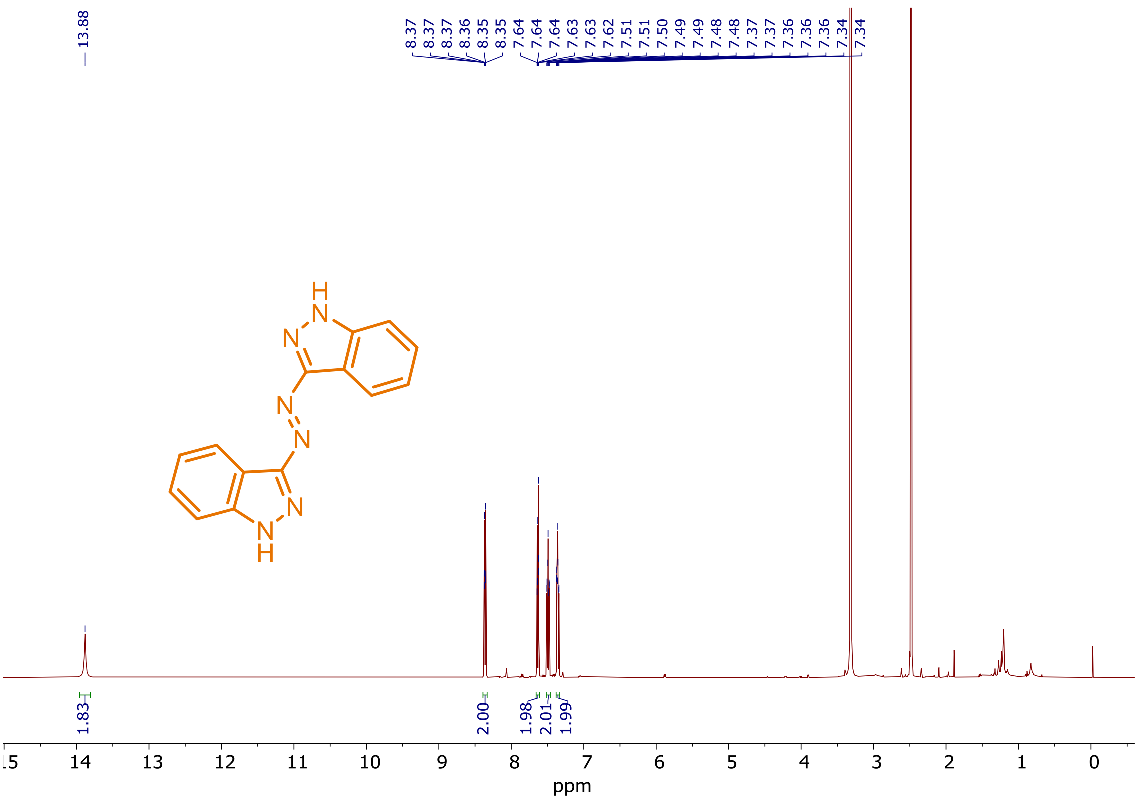

Figure S11. ^{1}H NMR of compound **7** (500 MHz, DMSO-d_6).

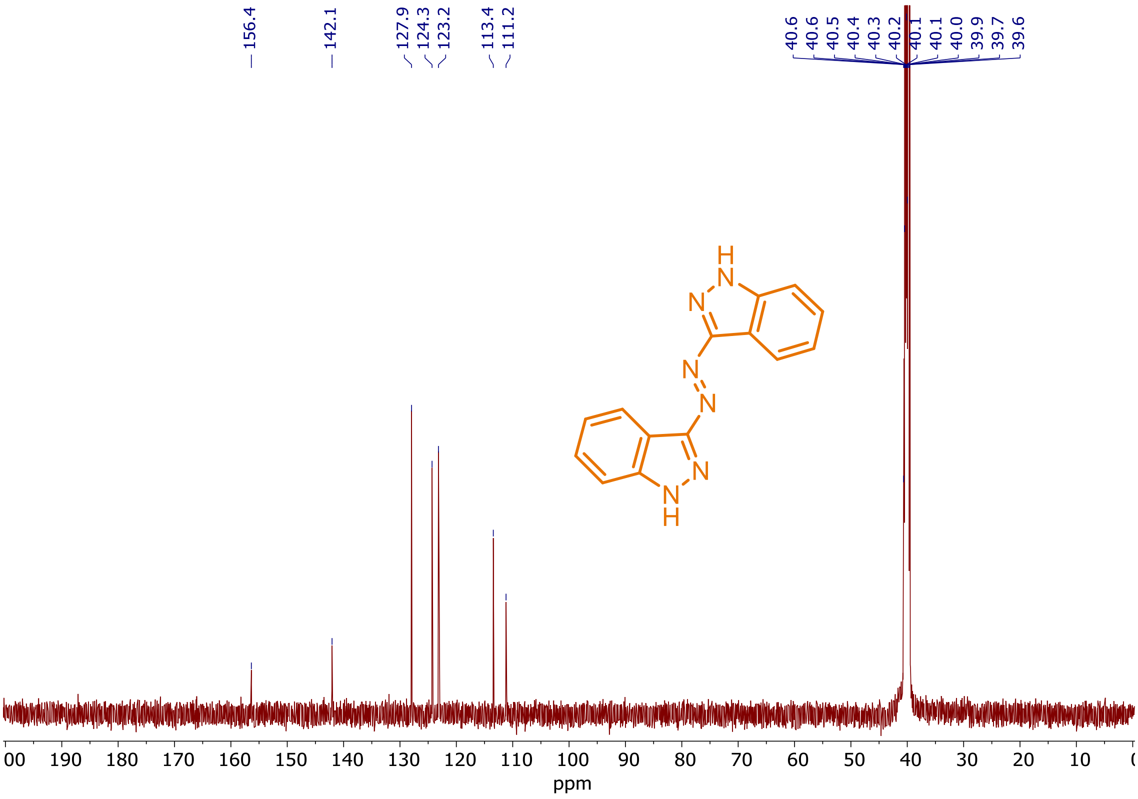

Figure S12. $^{13}C\{^{1}H\}$ NMR of compound **7** (126 MHz, DMSO-d_6).

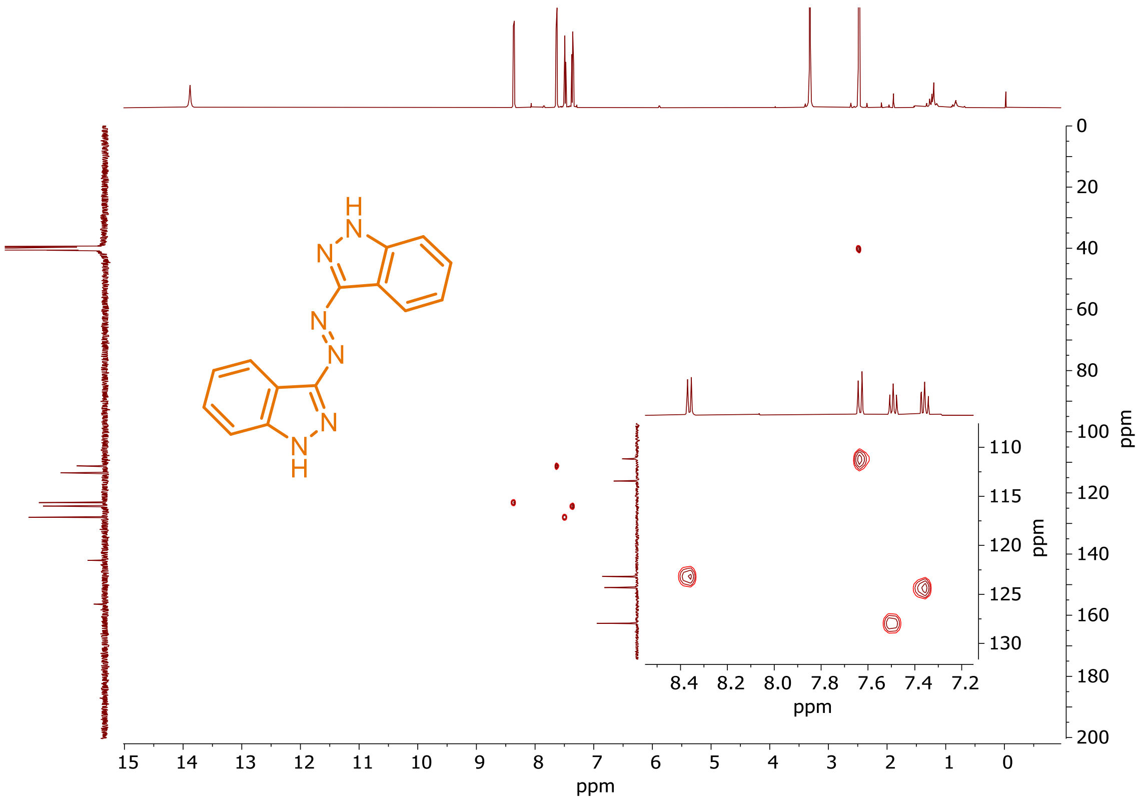

Figure S13. ^{1}H-^{13}C HSQC of compound **7** (500 MHz, DMSO-d_6).

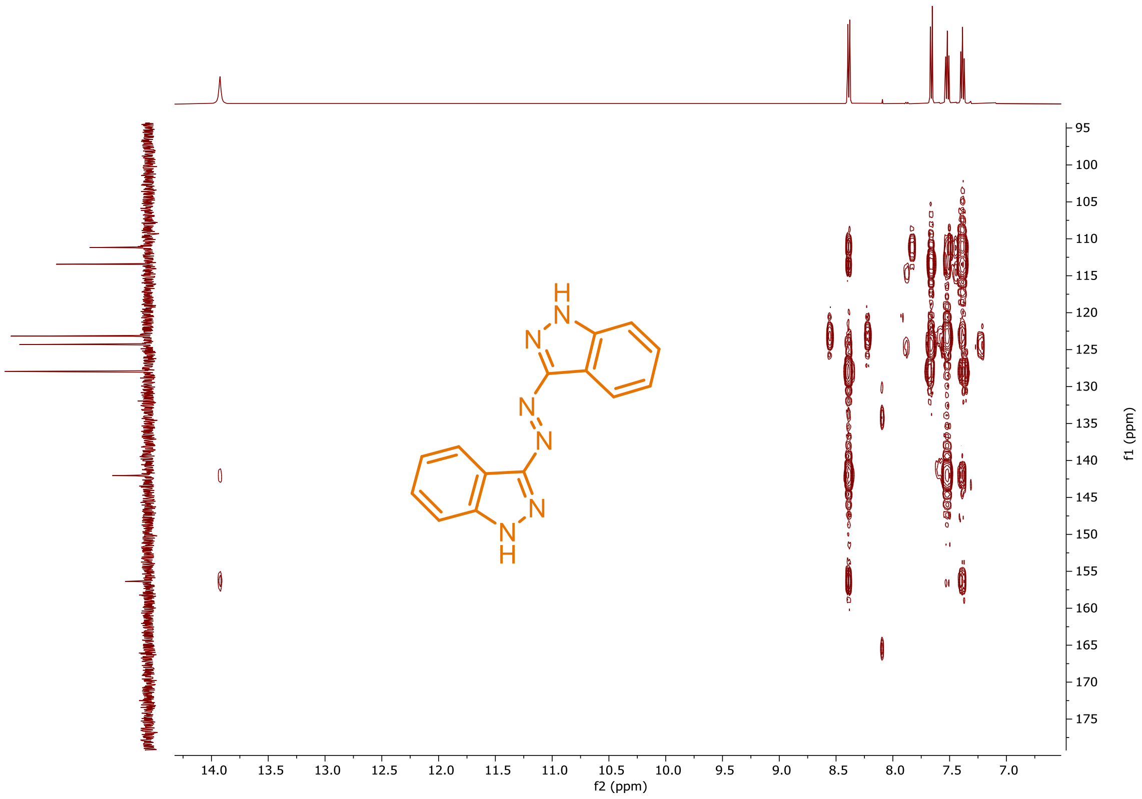

Figure S14. ^{1}H-^{13}C HMBC of compound **7** (500 MHz, DMSO-d_6).

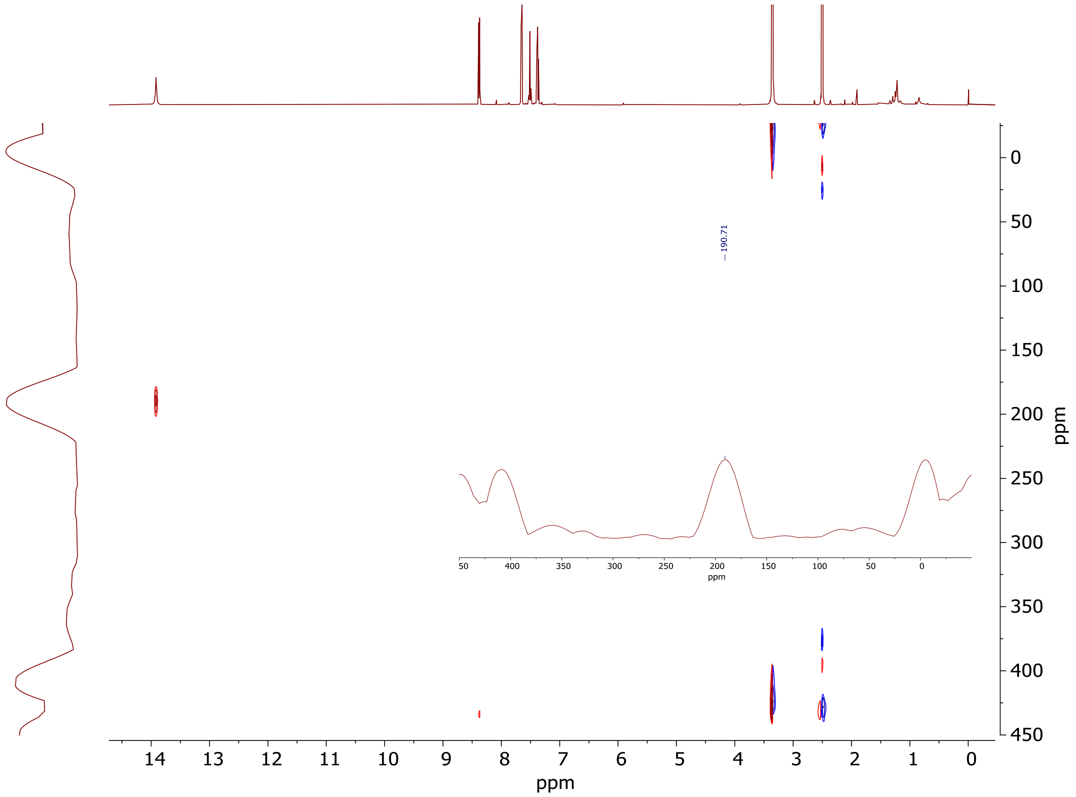

Figure S15. ^{1}H-^{15}N HSQC of compound **7** and HSQC projection (inset) (500 MHz, DMSO-d_6).

X-ray diffraction of compounds 6 and 7

Single-crystals of compounds **6** (red flake) and **7** (yellow/pale orange flake) were manually selected from the crystallization vial (Solution in DMF, not dried, in open air). A suitable single-crystal was mounted on a glass fiber with the help of silicon grease.

Data were collected at 150(2) K on a RIGAKU XtaLAB Synergy-i instrument equipped with a Mo Kα (λ = 0.71073 Å) PhotonJet-i micro source and a HyPix3000 detector. The data was controlled by the CrysAlisPro software (Y. Oxford Diffraction Ltd, England, 2022, CrysAlis PRO, Rigaku V1.171.142.173a) and an Oxford Cryosystems Series 800 cryostream was used. The diffraction images were processed using CrysAlisPro software (Y. Oxford Diffraction Ltd, England, 2022, CrysAlis PRO, Rigaku V1.171.142.173a.). The data was corrected for absorption using the multi-scan absorption correction with spherical harmonics implemented in the SCALE3 ABSPACK scaling algorithm. The structures were solved using direct methods with SHELXT 2014/5 and refined using the weighted full-matrix least-squares method on F^2 with SHELXL2018/3. Anisotropic thermal parameters were used to refine all non-hydrogen atoms. Molecular diagrams were created using Mercury software.

Hydrogen atoms bound to carbon were located at their idealized positions using appropriate *HFIX* instructions in SHELXL (*43* for the aromatic and vinylic, *23* for the $–CH_2–$ moieties and *13* for the chiral tertiary carbon atoms) and included in subsequent refinement cycles in riding-motion approximation with isotropic thermal displacements parameters (U_{iso}) fixed at 1.2 times U_{eq} of the atom to which they are attached.

Crystallographic data for the structures reported in this article have been deposited with the Cambridge Crystallographic Data Centre as supplementary publication No. CCDC 2514535-2514536. Copies of the data can be obtained free of charge on application to CCDC, 12 Union Road, Cambridge CB2 2EZ, U.K. Fax: (+44) 1223 336033. E-mail: deposit@ccdc.cam.ac.uk.

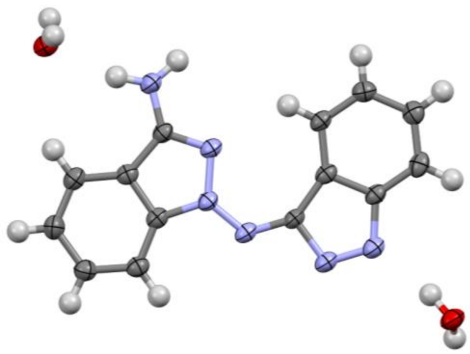

The single crystal of compound **6** was obtained by slow evaporation of a DMF solution in open air. A suitable crystal was selected for single-crystal X-ray diffraction analysis. The crystallographic data are summarized as follows:

CCDC number	2514535
Empirical formula	$C_{14} H_{10} N_6$, 2(H_2O)
Formula weight	298.31
Temperature [K]	150.00(10)
Crystal system	Triclinic
Space group (number)	P-1
a [Å]	6.7140(8)
b [Å]	9.8632(8)
c [Å]	10.7886(15)
α [°]	77.725(9)
β [°]	76.363(11)
γ [°]	86.482(8)
Volume [Å^3]	678.38(14)
Z	2
ρ_{calc} [gcm^{-3}]	1.460
μ [mm^{-1}]	0.104
F(000)	312.0
Crystal size [mm^3]	0.10 × 0.25 × 0.50
Radiation	Mo Kα (λ = 0.71073)
2θ range [°]	3.968 to 54.296
Index ranges	-8 ≤ h ≤ 8, -12 ≤ k ≤ 12, -13 ≤ l ≤ 13
Reflections collected	13915
Independent reflections	2866 [R_{int} = 0.0554, R_{sigma} = 0.0729]
Data / Restraints / Parameters	2866/0/223
Goodness-of-fit on F^2	1.017
Final R indexes [I>=2σ (I)]	R_1 = 0.0556, wR_2 = 0.1194
Final R indexes [all data]	R_1 = 0.1156, wR_2 = 0.1439
Largest diff. peak/hole / e Å^{-3}	0.305/-0.194

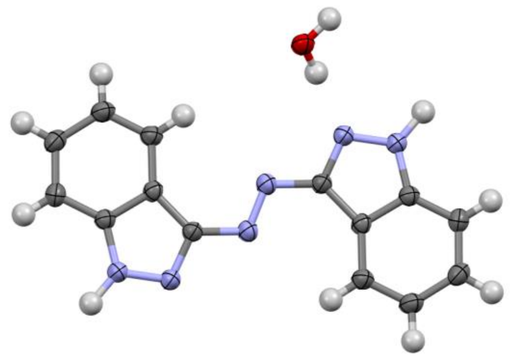

The single crystal of compound **7** was obtained by slow evaporation of a DMF solution in open air. A suitable crystal was selected for single-crystal X-ray diffraction analysis. The crystallographic data are summarized as follows:

CCDC number	2514536
Empirical formula	$C_{14} H_{10} N_6$, H_2O
Formula weight	280.30
Temperature [K]	150.00(10)
Crystal system	Orthorhombic
Space group (number)	P c c n
a [Å]	13.6494(7)
b [Å]	11.1362(5)
c [Å]	8.8135(4)
α [°]	90
β [°]	90
γ [°]	90
Volume [Å^3]	1339.67(11)
Z	4
ρ_{calc} [gcm^{-3}]	1.390
μ [mm^{-1}]	0.095
F(000)	584.0
Crystal size [mm^3]	0.05 × 0.40 × 0.60
Radiation	Mo Kα (λ = 0.71073)

2θ range [°]	4.720 to 50.484
Index ranges	$-17 \leq h \leq 17$, $-14 \leq k \leq 14$, $-11 \leq l \leq 10$
Reflections collected	10828
Independent reflections	1553 [R_{int} = 0.0476, R_{sigma} = 0.0373]
Data / Restraints / Parameters	1553/0/104
Goodness-of-fit on F^2	1.099
Final R indexes [I>=2σ (I)]	R_1 = 0.0483, wR_2 = 0.1298
Final R indexes [all data]	R_1 = 0.0677, wR_2 = 0.1409
Largest diff. peak/hole / e $Å^{-3}$	0.229/-0.219

Mechanistic analysis of deprotonation sequence

For the mechanistic study of deprotonation sequence, the ground state geometries and transition states (TS) were optimized using method/basis set: ucam-b3lyp/aug-cc-pvtz with SMD solvent model for THF. The empirical dispersion D3 with the Becke-Johson damping D3(BJ) has been included. Vibrational frequencies confirmed minima geometries (no imaginary frequencies) and transition states (single imaginary frequency). All calculations were performed with Gaussian 16 C0.1.

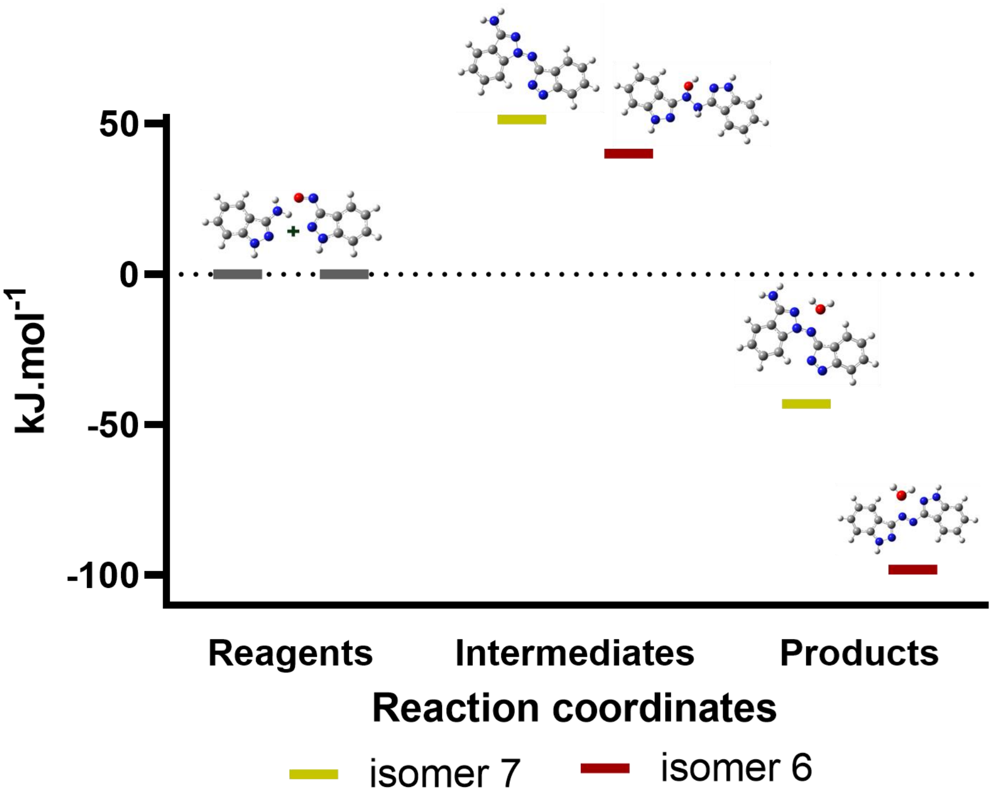

Figure S16. Global mechanistic view of isomer **6** (red) and **7** (yellow) pathway considering compound **5** and nitrosoarene (grey) as the starting point (0 kJ.mol^{-1}) with product values including H_2O molecule.

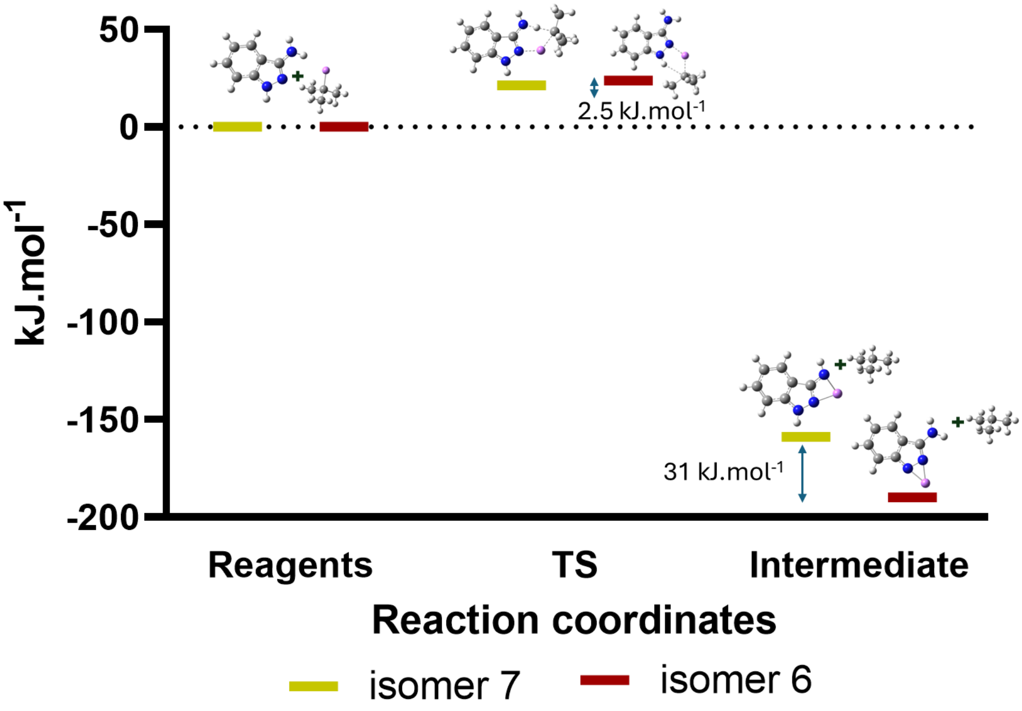

Figure S17. First deprotonation step of compound **5** reaction with *t*-BuLi tracking primary amine (yellow) or imidazole (red) to yield isomer **6** and **7** single deprotonation intermediates and *t*-Butyl.

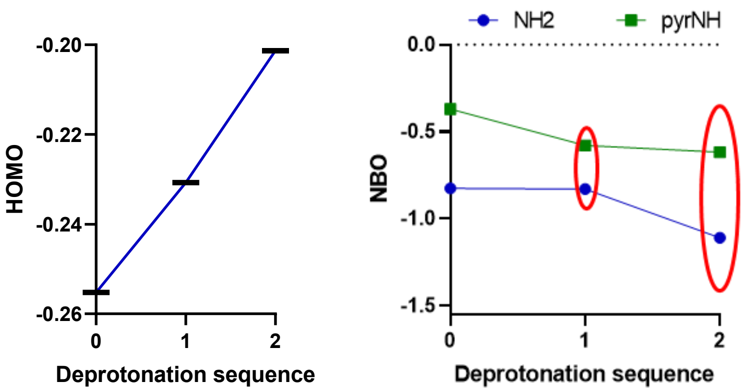

Figure S18. HOMO and localized NBO charge (in Hartree) progression of primary amine (blue) and pyrazole (green) during deprotonation sequence with *t*-BuLi. Highlight (red) for the lowest and higher NBO charge gaps between primary amine and pyrazole amine.

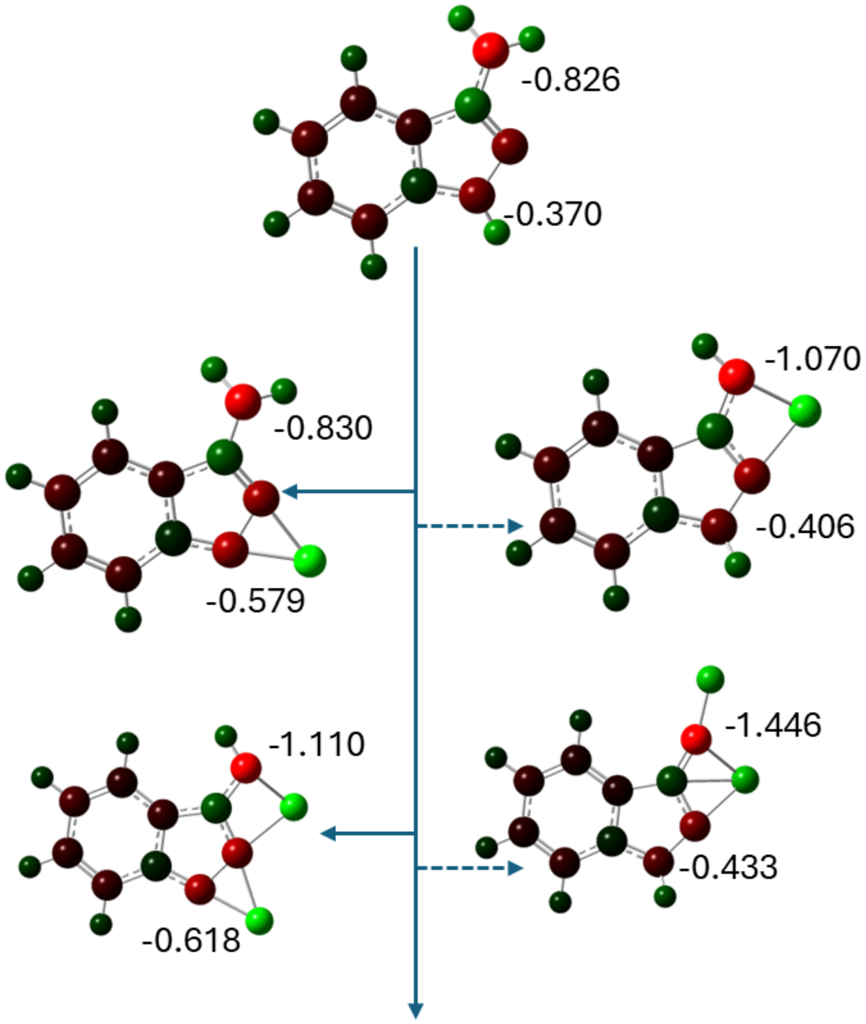

Figure S19. Localized NBO charges of each intermediate in the deprotonation sequence as an indication of nucleophilicity progression.

Table S1. Electronic energy (Hartree), number of imaginary frequencies and cartesian coordinates (Å) of **6** and **7** at B3LYP/6-311++G(d,p)/D3(BJ) computational level.

**6**	**7**
Electronic Energy= -868.224787137	Electronic Energy= -868.242793667
NIMAG= 0	NIMAG= 0
C,4.9441656689,0.0156032136,0.0935903182	N,-0.6116558166,-0.1617588087,0.
C,3.5937158302,-0.0954899938,-0.2666187886	N,0.6116558166,0.1617588087,0.
C,2.6146266729,0.5754029685,0.4713719371	C,-0.8241977299,-1.5313915299,0.
C,3.0265555071,1.335170393,1.5580084916	C,0.0914453926,-2.6472180254,0.
C,4.3884338736,1.4286689633,1.9040973135	C,-0.7518408357,-3.7816615653,0.
C,5.3749466133,0.773357306,1.1783136754	N,-2.0367952287,-3.2921463204,0.
C,3.5998423357,-0.966558136,-1.4238011188	N,-2.0870769321,-1.9495510113,0.
H,1.56571214,0.5158656798,0.20556213	H,-2.90038789,-3.8094462051,0.
H,2.2924661118,1.8682003912,2.1494969444	C,0.8241977299,1.5313915299,0.
H,4.6741571369,2.0307518183,2.7583181928	C,-0.0914453926,2.6472180254,0.
H,6.424738927,0.8380376582,1.4294070706	C,0.7518408357,3.7816615653,0.
N,2.5042007561,-1.3288234445,-2.1603593553	N,2.0367952287,3.2921463204,0.
H,2.6380830007,-2.0898848122,-2.8096408158	N,2.0870769321,1.9495510113,0.
H,1.6170217821,-1.3381151534,-1.682140668	H,2.90038789,3.8094462051,0.
N,4.8162487798,-1.3500184997,-1.7473630943	C,1.4839817863,-2.8196214432,0.
N,5.6618378989,-0.7451540196,-0.825549637	C,-1.4839817863,2.8196214432,0.
N,6.961074723,-0.8583613346,-0.7434789971	C,-0.2556518731,-5.0891357575,0.
C,7.7252361825,-1.5313256997,-1.5466577454	C,0.2556518731,5.0891357575,0.
C,7.6426847678,-2.3577494794,-2.7525411198	C,-1.9811144516,4.1130421362,0.
C,8.9885338786,-2.7082947951,-2.9753020887	H,-3.0525363161,4.2735836593,0.
C,6.6674107977,-2.814575885,-3.6383956253	C,1.9811144516,-4.1130421362,0.
C,9.3948230618,-3.4982421105,-4.0393778656	H,3.0525363161,-4.2735836593,0.
C,7.0678995761,-3.6095010443,-4.7141895952	C,-1.122237704,5.232200562,0.
H,5.6324289395,-2.5540699756,-3.4886558024	H,-1.5511911691,6.227450643,0.
C,8.4097521289,-3.9510364121,-4.9163871926	C,1.122237704,-5.232200562,0.
H,10.4412942856,-3.7433643654,-4.1721975205	H,1.5511911691,-6.227450643,0.
H,6.3192670147,-3.9700865054,-5.4103945928	H,2.1348438061,-1.9575283427,0.
H,8.6835417657,-4.5705041974,-5.7623718591	H,-2.1348438061,1.9575283427,0.
N,9.1124696205,-1.4666102574,-1.1687642552	H,-0.9138896836,-5.9495182641,0.
N,9.8204502029,-2.1374892806,-1.9790631959	H,0.9138896836,5.9495182641,0.
